# Microglial cannabinoid receptor type II stimulation improves cognitive impairment and neuroinflammation in Alzheimer’s disease mice by controlling astrocyte activation

**DOI:** 10.1038/s41419-024-07249-6

**Published:** 2024-11-26

**Authors:** Akira Sobue, Okiru Komine, Fumito Endo, Chihiro Kakimi, Yuka Miyoshi, Noe Kawade, Seiji Watanabe, Yuko Saito, Shigeo Murayama, Takaomi C. Saido, Takashi Saito, Koji Yamanaka

**Affiliations:** 1 Department of Neuroscience and Pathobiology, Research Institute of Environmental Medicine, Nagoya University, Aichi, 464-8601 Japan; 2 Department of Neuroscience and Pathobiology, Nagoya University Graduate School of Medicine, Aichi, 466-8550 Japan; 3 Medical Interactive Research and Academia Industry Collaboration Center, Research Institute of Environmental Medicine, Nagoya University, Aichi, 464-8601 Japan; 4 Brain Bank for Aging Research (Neuropathology), Tokyo Metropolitan Geriatric Hospital and Institute of Gerontology, Tokyo, 173-0015 Japan; 5 Brain Bank for Neurodevelopmental, Neurological and Psychiatric Disorders, United Graduate School of Child Development, Osaka University, Osaka, Japan; 6 Laboratory for Proteolytic Neuroscience, RIKEN Center for Brain Science, Saitama, 351-0198 Japan; 7 Department of Neurocognitive Science, Institute of Brain Science, Nagoya City University Graduate School of Medical Sciences, Aichi, 467-8601 Japan; 8 Institute for Glyco-core Research (iGCORE), Nagoya University, Aichi, Japan; 9 Center for One Medicine Innovative Translational Research (COMIT), Nagoya University, Aichi, Japan; 10 Research Institute for Quantum and Chemical Innovation, Institutes of Innovation for Future Society, Nagoya University, Aichi, Japan

**Keywords:** Microglia, Alzheimer's disease

## Abstract

Alzheimer’s disease (AD) is the most common form of dementia and is characterized by the accumulation of amyloid β (Aβ) and phosphorylated tau. Neuroinflammation, mainly mediated by glial activation, plays an important role in AD progression. Although there is growing evidence for the anti-neuroinflammatory and neuroprotective effects of the cannabinoid system modulation, the detailed mechanism remains unclear. To address these issues, we analyzed the expression levels of cannabinoid receptor type II (*Cnr2/Cb2*) in *App*^*NL-G-F/NL-G-F*^ mice and human AD precuneus, which is vulnerable to amyloid deposition in AD, and the effects of JWH 133, a selective CB2 agonist, on neuroinflammation in primary glial cells and neuroinflammation and cognitive impairment in *App*^*NL-G-F/NL-G-F*^ mice. The levels of *Cnr2/Cb2* were upregulated in microglia isolated from the cerebral cortex of *App*^*NL-G-F/NL-G-F*^ mice. *CNR2* expression was also increased in RNAs derived from human precuneus with advanced AD pathology. Chronic oral administration of JWH 133 significantly ameliorated the cognitive impairment of *App*^*NL-G-F/NL-G-F*^ mice without neuropsychiatric side effects. Microglia and astrocyte mRNAs were directly isolated from the mouse cerebral cortex by magnetic-activated cell sorting, and the gene expression was determined by quantitative PCR. JWH 133 administration significantly decreased reactive astrocyte markers and microglial *C1q*, an inducer for the reactive astrocytes in *App*^*NL-G-F/NL-G-F*^ mice. In addition, JWH133 administration inhibited the expression of p-STAT3 (signal transducer and activator of transcription 3) in astrocytes in *App*^*NL-G-F/NL-G-F*^ mice. Furthermore, JWH 133 administration suppressed dystrophic presynaptic terminals surrounding amyloid plaques. In conclusion, stimulation of microglial CB2 ameliorates cognitive dysfunction in *App*^*NL-G-F/NL-G-F*^ mice by controlling astrocyte activation and inducing beneficial neuroinflammation, and our study has implications that CB2 may represent an attractive therapeutic target for the treatment of AD and perhaps other neurodegenerative diseases involving neuroinflammation.

## Introduction

Alzheimer’s disease (AD) is the most common form of dementia and is neuropathologically characterized by the accumulation of amyloid β (Aβ), phosphorylated tau, and neuronal loss [1]. Neuroinflammation is mediated by the activation of the innate immune system of the brain in response to inflammatory challenges, including misfolded protein aggregates that are often accumulated in neurodegenerative diseases [2, 3]. Microglia, resident innate immune cells in the central nervous system (CNS), are involved in immune surveillance and homeostasis in the CNS and are key players in mediating neuroinflammation. They play a critical role in sensing and clearing Aβ in AD [3–6].

Cannabinoids, whether plant-derived, synthetic, or endocannabinoids, have beneficial effects, such as anti-inflammatory and analgesic effects [7]. Cannabinoids interact mainly with two types of cannabinoid Gi/o-coupled receptors: cannabinoid receptor type I (CNR1; CB1) and type II (CNR2; CB2) [8]. The CB1 is widely expressed throughout the CNS, and is highly enriched in the presynaptic and axonal compartments of neurons, where it regulates neurotransmitter release and synaptic activity [9]. Therefore, CB1 agonists have adverse effects, such as motor impairment, amnesia, and changes in mood and anxiety in some cases [10]. In contrast, CB2, which is mainly expressed in peripheral immune cells and CNS microglia, modulates inflammation by regulating cell activation and cytokine release [11]. A relatively low expression of CB2 in neurons has also been reported [12]. Therefore, CB2 has attracted considerable attention as a potential new therapeutic target for neurological diseases [13–16]. Although several studies have shown that chronic administration of CB2 agonists ameliorates cognitive impairment and gliosis in AD models with *App* gene overexpression or Aβ injection [13, 15, 17–19], the detailed mechanisms of action of CB2 agonists as therapeutic agents in AD remain unclear. In addition, some studies have reported controversial results indicating that CB2 deletion ameliorates cognitive and learning deficits and pathologies in transgenic APP/PSEN1 mice and rTg4510 mice [20, 21]. Therefore, whether CB2 stimulation is beneficial for AD pathologies is still under debate.

In postmortem AD brains, CB2 expression has been reported to be upregulated in the frontal or temporal cortex, or hippocampus of patients with AD [20, 22, 23]. However, CB2 expression in the precuneus, which is vulnerable to Aβ deposition in preclinical AD, has not been investigated. The precuneus is located medially in the parietal lobe of the cerebral cortex, and it is a component of the default mode network, which is implicated in episodic memory retrieval, and displays high metabolic activity during the baseline resting state [24]. Amyloid positron emission tomography studies have shown that the precuneus is a brain region where Aβ accumulation preferentially starts in preclinical AD [25, 26]. Therefore, uncovering the alteration of CB2 expression in the precuneus is important to better understand the inflammatory response in amyloid pathologies.

To address these issues, we first performed a comparative analysis of CB2 gene expression in isolated microglia from *App*^*NL-G-F/NL-G-F*^ mice and the precuneus of the patients with AD. Subsequently, we examined whether chronic CB2 receptor stimulation confers neuroprotection and ameliorates cognitive dysfunction in mice with AD. Furthermore, the detailed gene expression of isolated glial cells in mice with AD was analyzed.

## Results

### *Cnr2/CNR2* mRNA expression is commonly upregulated in isolated microglia from *App*^*NL-G-F/NL-G-F*^ mice and human precuneus with AD pathology

**Fig. 1:**
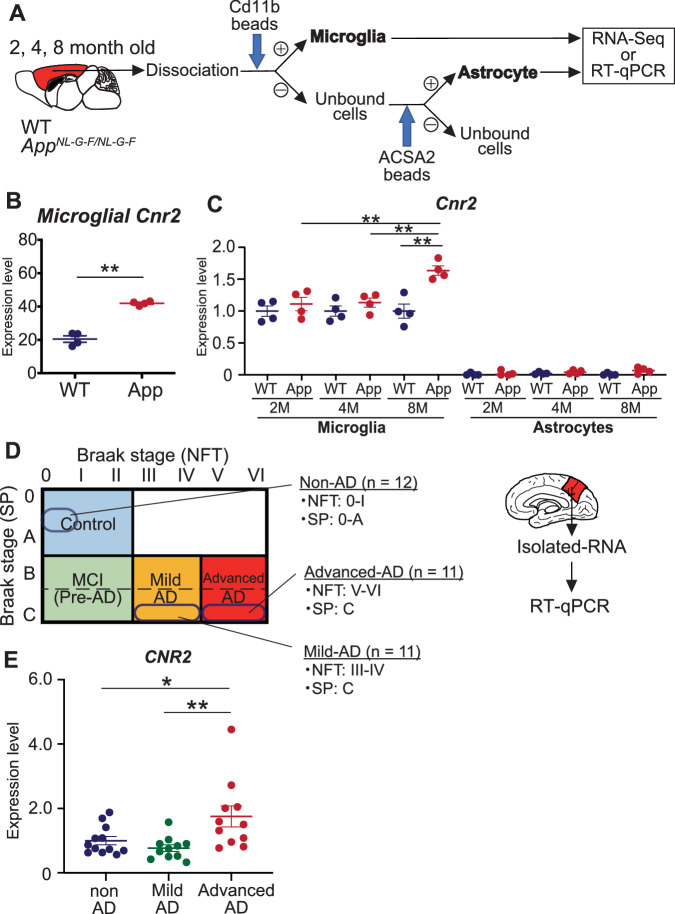
Expression of *Cb2/CB2* mRNA was commonly upregulated in microglia isolated from *App*^*NL-G-F/NL-G-F*^ mice and precuneus of the patients with AD. **A** Schematic overview of gene expression analysis of microglia and astrocytes isolated from *App*^*NL-G-F/NL-G-F*^. **B** *Cnr2* gene expression was analyzed using RNA sequencing (RNA-Seq) in isolated microglia (WT: *n* = 4 and *App*^*NL-G-F/NL-G-F*^: *n* = 4). WT, wild-type; App, *App*^*NL-G-F/NL-G-F*^. Data are presented as means ± standard error of the mean (SEM). **q < *0.01*. **C** Quantitative PCR analysis to determine the expression levels of *Cnr2* mRNA in glial cells isolated from 2, 4, and 8-month-old *App*^*NL-G-F/NL-G-F*^ mice and WT mice. Data are presented as means ± SEM. N.D.: not detectable. **p < *0.01 (two-way ANOVA)*. **D** Human brain samples were selected for analysis based on the Braak staging as follows: control brain (non-AD) defined as Braak stage (senile plaque: SP): 0–A, Braak stage (neurofibrillary tangle: NFT): 0–I; mild-AD brain defined as Braak stage (SP): C, Braak stage (NFT): III–IV; and advanced AD brain defined as Braak stage (SP): C and Braak stage (NFT): V-VI. Schematic overview of the gene expression analysis of *CNR2* in the precuneus of non-AD (*n* = 12), mild-AD (*n* = 11), and advanced-AD (*n* = 11). **E** Quantitative PCR analysis to determine the expression levels of *CNR2* mRNA in the precuneus of non-AD (*n* = 12), mild-AD (*n* = 11), and advanced-AD (*n* = 11). Data are presented as means ± SEM. *p < 0.05, **p < *0.01 (two-way ANOVA)*.

Although accumulating evidence suggests that Cnr2/CNR2 is expressed at high levels in the brain tissue of AD patients and AD mice [20, 22, 23, 27], very few studies have determined the Cnr2/CNR2 gene expression in isolated glial cells from AD model mice and human AD precuneus. Therefore, we first analyzed the *Cnr2* expression levels in cortical microglia isolated from *App*^*NL-G-F/NL-G-F*^ mice, which exhibit cognitive decline with amyloid pathology and neuroinflammation (Fig. 1A) [28]. We found that the expression level of *Cnr2* mRNA was significantly increased in isolated microglia from 8-month-old *App*^*NL-G-F/NL-G-F*^ mice compared with that in WT mice (Fig. 1B). To clarify the cell type that expresses *Cnr2* during AD progression, we measured the mRNA levels of *Cnr2* using isolated microglia and astrocytes in the cerebral cortices of *App*^*NL-G-F/NL-G-F*^ mice at 2, 4, and 8 months of age using RT-qPCR. We found that *Cnr2* was expressed in microglia and significantly upregulated in 8-month-old *App*^*NL-G-F/NL-G-F*^ mice (Fig. 1C).

Although some studies have shown that the expression of *CNR2* was increased in the frontal cortex of patients with AD [23, 29], few studies have focused on the precuneus, the brain region where Aβ accumulation preferentially starts in preclinical AD. We examined the expression level of *CNR2* mRNA in the precuneus. Data for Braak neuropathological stages, neuropathological diagnosis, Clinical Dementia Ratings (CDR), APOE genotypes, and RNA quality of human brain samples are summarized in Table 1. Postmortem brain samples were selected based on the Braak neuropathological staging, as follows: 12 non-AD brains were graded 0–A for senile plaque (SP) and 0–I for NFT, 11 mild-AD brains were graded C for senile plaque (SP) and III–IV for NFT, and 11 advanced-AD brains were graded C for SP and V-VI for NFT (Fig. 1D and Table 1). While no differences in the expression of *CNR2* were observed between non-AD and mild-AD brains, it was significantly increased in the precuneus of patients with advanced-AD compared with that in non-AD brains (Fig. 1E). These results indicate that CB2 is involved in AD progression and is commonly upregulated in the human AD brain and microglia isolated from *App*^*NL-G-F/NL-G-F*^ mice.

**Table 1:** Clinical and neuropathological data of human subjects.

Subject number	RIN	Age	Sex	Braak senile plaque stage	Braak NFT stage	Grain stage	*APOE genotype*	CDR	Neuropathological diagnosis
non-AD 1	7.93	83	M	0	1	0	33	NA	Unremarkable
non-AD 2	7.4	80	F	1	1	0.5	33	1	Asymptomatic lacunar infarction
non-AD 3	8.3	79	F	1	1	0	33	1	Asymptomatic lacunar infarction
non-AD 4	8.7	70	M	1	0.5	0	34	0	Unremarkable
non-AD 5	7.73	81	M	1	0	0	23	0	Unremarkable
non-AD 6	7.63	82	M	1	1	0	33	0	Unremarkable
non-AD 7	7.2	75	F	1	1	0.5	33	0	Unremarkable
non-AD 8	8.9	73	M	1	0	0.5	33	0	Unremarkable
non-AD 9	9	75	M	1	1	3	34	0	AGD
non-AD 10	7.6	71	F	0	1	1	33	0	Unremarkable
non-AD 11	8.1	82	F	1	1	2	33	0	Unremarkable
non-AD 12	9.5	86	M	0	1	0.5	34	0	CVD
Mild-AD 1	7.6	78	M	3	3	0	33	3	Early AD
Mild-AD 2	7.7	76	M	3	3	1	33	NA	Alzheimer change
Mild-AD 3	6.8	81	F	3	4	1	33	1	AD
Mild-AD 4	8.43	70	M	3	3	1	44	1	AD
Mild-AD 5	8	82	M	3	3	2/1	34	NA	Alzheimer change, CVD
Mild-AD 6	8	82	M	3	3	0.5	34	0.5	CVD, Alzheimer change, CSH
Mild-AD 7	8.1	85	M	3	3	2	33	0	CVD, CSH
Mild-AD 8	8.2	86	F	3	3	2	33	NA	CVD
Mild-AD 9	7.9	94	M	3	3	0	33	1	AD
Mild-AD 10	8	90	F	3	3	2	33	0	AGD
Mild-AD 11	8.2	93	M	3	3	2	23	2	CVD, CSH
Advanced-AD 1	7.7	74	M	3	5	1	34	1	AD/ CVD
Advanced-AD 2	8.1	82	F	3	6	2	33	3	AD/ CVD
Advanced-AD 3	7.6	82	M	3	5	1/1	33	NA	AD, ILBD,SDH,CVD
Advanced-AD 4	7.8	83	M	3	5	０	34	3	AD
Advanced-AD 5	8.7	85	M	3	5	2	23	3	AD/ AA
Advanced-AD 6	8.8	85	M	3	5	0.5/0	34	NA	AD
Advanced-AD 7	8.47	86	F	3	5	0	34	3	AD
Advanced-AD 8	8.47	87	F	3	5	0	34	3	AD
Advanced-AD 9	8.5	81	M	3	6	0	33	1or 2	AD
Advanced-AD 10	7.8	78	M	3	5	1	33	2	AD/ CVD/ CSH
Advanced-AD 11	8.7	87	M	3	6	0	34	3	AD

*CSH* Chronic subdural hematoma, *AGD* Agyrophilic grain disease, *AD* Alzheimer’s disease, *CVD* Cerebrovascular disease, *ILBD* Incidental Lewy body disease, *SDH* Subdural hematom, *AA* Amyloid angiopathy, Braak senile plaque stage: 1=A, 2=B, 3=C.

### Microglial CB2 stimulation suppresses astrocyte activation in vitro

**Fig. 2:**
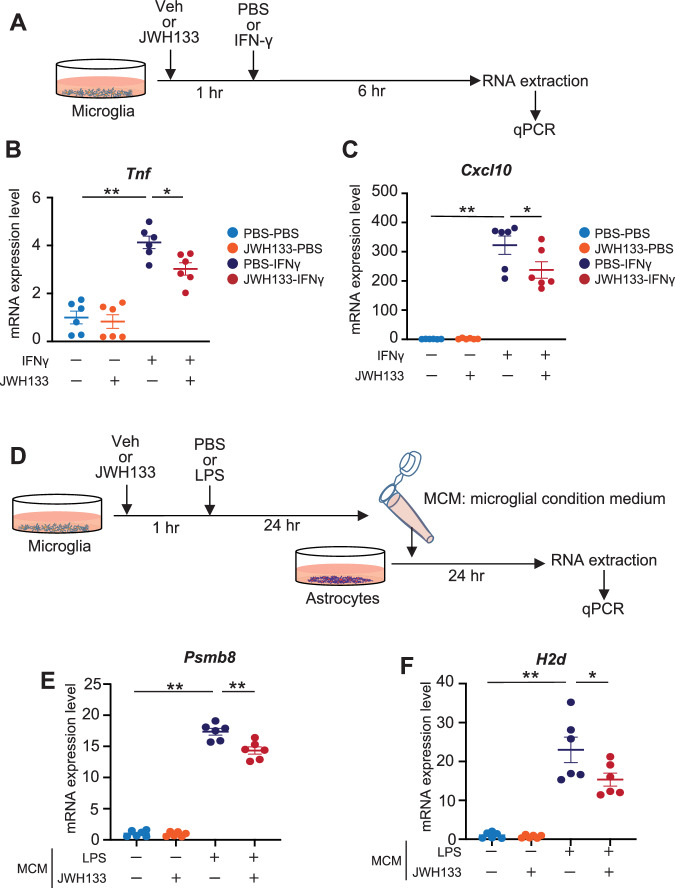
Microglial CB2 stimulation suppresses both microglial and astrocytic activation in vitro. **A** A schematic protocol for JWH 133 treatment in primary microglia. **B**, **C** Expression levels of mRNAs in JWH 133-treated primary microglia determined by quantitative PCR. Relative expression levels for *Tnf* (**B**) and *Cxcl10* (**C**) were determined and are presented as means ± SEM. *n* = 6, each. *p < 0.05, **p < *0.01 (two-way ANOVA)*. **D** A schematic protocol for microglial condition medium (MCM) treatment of primary astrocytes. **E**, **F** Expression levels of mRNAs in MCM-treated primary astrocytes determined by quantitative PCR. Relative expression levels for *Psmb8* (**E**) and *H2d* (**F**) were determined and are presented as means ± SEM. *n* = 6, each. *p < 0.05, **p < 0.01 *(two-way ANOVA)*.

To investigate the effects of microglial CB2 stimulation on inflammation, we examined the effects of JWH 133, a selective CB2 agonist (Ki = 677 nM for CB1 and 3.4 nM for CB2) [30], on the expression of inflammatory cytokines/chemokines in IFN-γ-activated microglia (Fig. 2A). We found that the expression of *Tnf* and *Cxcl10* was significantly increased in IFN-γ-activated microglia, whereas JWH 133 pretreatment significantly suppressed the expression of these cytokines/chemokines (Fig. 2B, C). Since activated microglia can induce reactive astrocytes [31], we investigated the possible contribution of microglial CB2 in astrocytic activation by analyzing the primary cultured astrocytes in microglia-conditioned medium (MCM) from microglia that were pre-exposed to Veh / JWH 133 or PBS / LPS (Fig. 2D). We found that Veh/LPS-activated MCM strongly induced reactive astrocytic markers such as proteasome 20S subunit beta 8 (*Psmb8*) and major histocompatibility complex (MHC) class I (*H2d*), whereas JWH 133/LPS-activated MCM significantly suppressed the expression of these markers (Fig. 2E, F). These results suggest that microglial CB2 stimulation by JWH 133 suppresses the proinflammatory phenotype in primary astrocytes, likely through controlling microglial activation.

### JWH 133 administration ameliorates the cognitive impairment in *App*^*NL-G-F/NL-G-F*^ mice

**Fig. 3:**
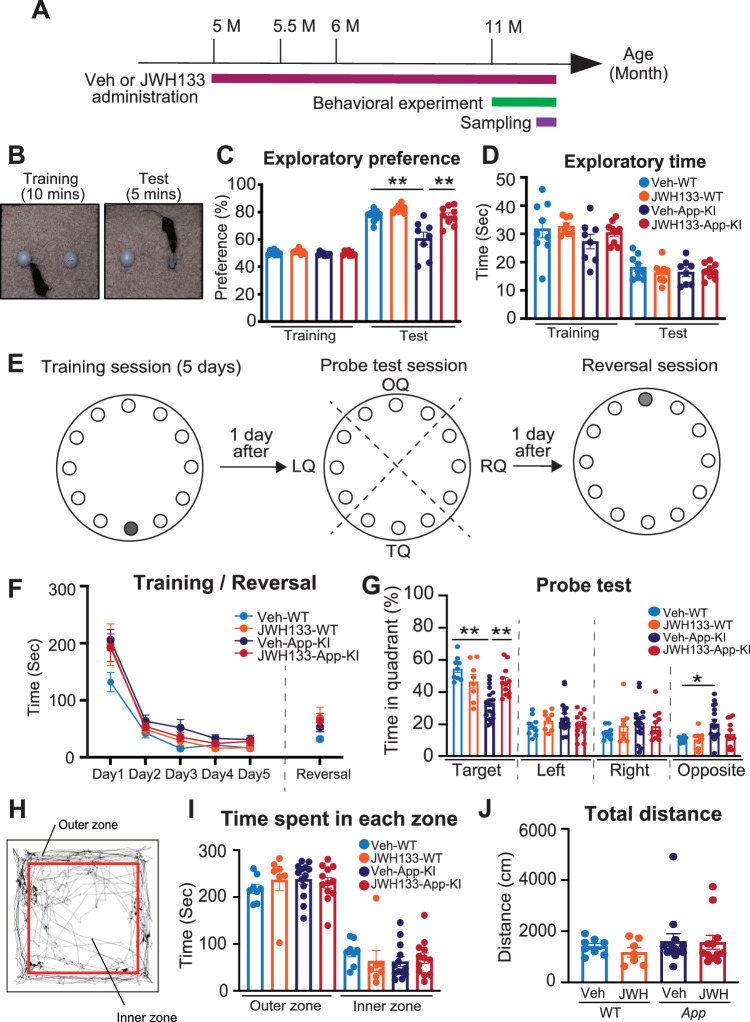
JWH 133 administration ameliorated the cognitive impairments in *App*^*NL-G-F/NL-G-F*^ mice. **A** Experimental timeline for JWH 133 administration and analysis of *App*^*NL-G-F*^ mice. **B** An experimental protocol for the novel object recognition test. **C**, **D** Effects of JWH 133 on performance in the novel object recognition test in WT and *App*^*NL-G-F/NL-G-F*^ mice. Exploratory preference (%) and time are plotted as means ± SEM [Veh-WT (*n* = 9), JWH 133-WT (*n* = 8), Veh-*App*^*NL-G-F/NL-G-F*^ (*n* = 9), JWH 133-*App*^*NL-G-F/NL-G-F*^ (*n* = 9)]. *p < 0.05 and **p < *0.01 (two-way ANOVA)*. **E** A schematic protocol for the Barnes maze test. In the training session (**E** left), one hole indicated as black was designated as the target hole with an escape box. A probe test was performed 1 day after the last training session, where the escape box was hidden (**E** middle). TQ: target quadrant; OQ: opposite quadrant; RQ: right quadrant; LQ: left quadrant. In the reversal session (**E** right), the target hole (gray) was relocated to the position opposite to the original position 1 day after the probe test. **F**, **G** Effects of JWH 133 on the performance of WT and *App*^*NL-G-F/NL-G-F*^ mice in the Barnes maze test. Values are presented means ± SEM [Veh-WT (*n* = 9), JWH 133-WT (*n* = 8), Veh-*App*^*NL-G-F/NL-G-F*^ (*n* = 15), JWH 133-*App*^*NL-G-F/NL-G-F*^ (*n* = 10)]. *p < 0.05 and **p < *0.01 (training: repeated measures three-way ANOVA, Probe test and Reversal: two-way ANOVA)*. **H** Image showing the travel path recorded for 5 min in the open field test arena. **I**, **J** Locomotor activity and anxiety were evaluated in an open field test as the time spent in each zone (**I**) and total distance traveled (**J**). Values are presented as means ± SEM. [Veh-WT (*n* = 8), JWH-WT (*n* = 7), Veh-*App*^*NL-G-F/NL-G-F*^ (*n* = 13), and JWH-*App*^*NL-G-F/NL-G-F*^ (*n* = 12)]. *(two-way ANOVA)*.

Based on the results of in vitro experiments (Fig. 2), we hypothesized that the JWH 133 ameliorates cognitive impairment in *App*^*NL-G-F/NL-G-F*^ mice by regulating neuroinflammation. Previous studies have reported that *App*^*NL-G-F/NL-G-F*^ mice show memory impairments as early as 6 months [32, 33]. However, other studies have reported that these mice show intact memory up to 10 months of age [34, 35]. In this study, to investigate whether *App*^*NL-G-F/NL-G-F*^ mice exhibit memory deficits and the effect of JWH 133 at 11 months of age, we administered JWH 133 or its vehicle to WT and *App*^*NL-G-F/NL-G-F*^ mice for 6 months, and then these animals were subjected to novel object recognition tests, open field test, and Barnes maze test during the treatment (Fig. 3A). The impairment in object recognition memory in *App*^*NL-G-F/NL-G-F*^ mice was significantly improved by repeated administration of JWH 133. However, the drug had negligible effects on object recognition memory in WT mice (Fig. 3B, C). No significant differences in the total time required to explore the two objects during the test session among the four groups of mice (Fig. 3B, D) or during the training sessions (Fig. 3B–D) were observed among the four groups of mice. Furthermore, we analyzed the effects of JWH 133 on spatial memory in *App*^*NL-G-F/NL-G-F*^ mice. In our experiments, the mice were asked to acquire the spatial location of a target hole that was connected to a dark escape box during the training session (Fig. 3E [left]). One day after the fifth session of the training, a probe test was performed without an escape box to investigate whether the mice had learned the location of the target hole using extra-maze cues (Fig. 3E [middle]). To further assess cognitive flexibility, the mice were subjected to the reversal learning task (five sessions) 1 day after the probe test (Fig. 3E [right]). We found that Veh-/JWH 133-administered WT and *App*^*NL-G-F/NL-G-F*^ mice performed equally well in learning the target hole in the Barnes maze test (Fig. 3F). In the probe test, Veh-administered *App*^*NL-G-F/NL-G-F*^ mice exhibited impaired preference for the target quadrant containing the target hole (Fig. 3G). JWH 133 administration substantially improved the performance of *App*^*NL-G-F/NL-G-F*^ mice in the probe test (Fig. 3G). In the reversal learning session (Fig. 3E [right]), each group showed similar performance levels in the reversal learning task (Fig. 3F).

CB2 is weakly expressed in the dopaminergic neurons and regulates dopamine-related behavior [36, 37]. Therefore, we evaluated the neuropsychiatric side effects of CB2 stimulation such as anxiety and locomotor activity using the open field test. No significant differences in the time spent in each zone among each group (Fig. 3H, I), or in the total distance traveled (Fig. 3H, J) were observed between the groups. These results indicate that JWH 133 ameliorates cognitive impairment in *App*^*NL-G-F/NL-G-F*^ mice without neuropsychiatric side effects.

### Chronic oral administration of JWH 133 ameliorates neuroinflammation in *App*^*NL-G-F/NL-G-F*^ mice

**Fig. 4:**
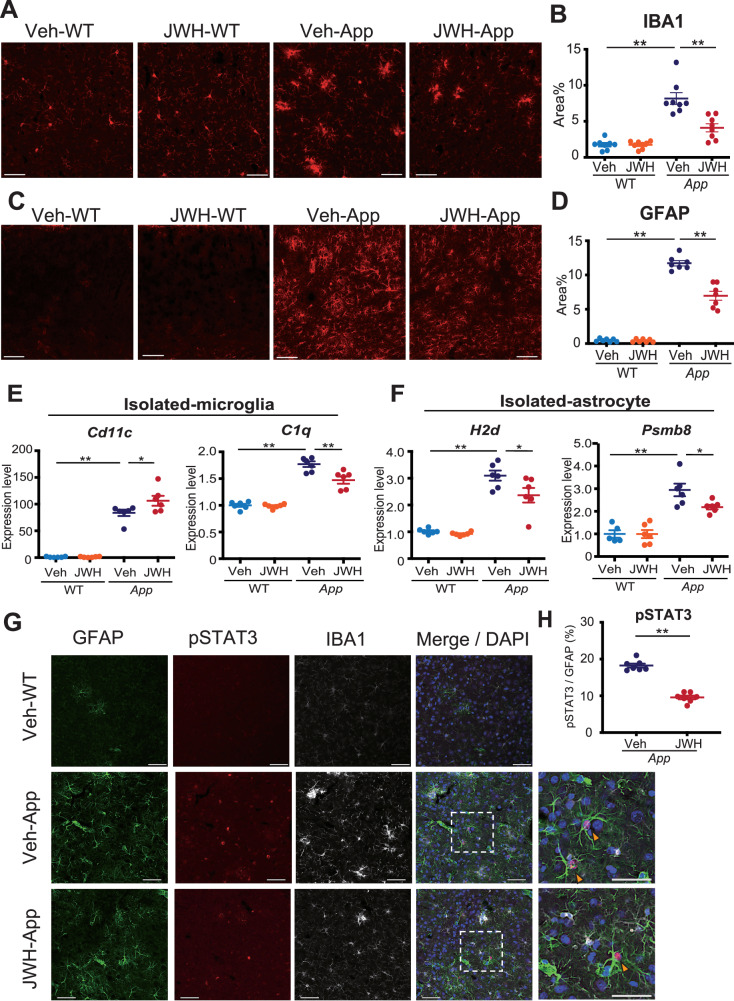
Chronic oral administration of JWH 133 ameliorates neuroinflammation in *App*^*NL-G-F/NL-G-F*^ mice. **A**–**D** Representative immunofluorescent images demonstrating the expression of IBA1 (red, **A**) or GFAP (red, **C**) in the cerebral cortices of Veh-/JWH 133-administered WT (left panels) and *App*^*NL-G-F/NL-G-F*^ mice (right panels) at 12 months old. Scale bars: 50 μm. Percentages of the area immunopositive for IBA1 (**B**) or GFAP (**D**) are quantified. Values are presented as means ± SEM (**B** and **D**). [*n* = 7–8. Seven sections per mouse were quantified.] *p < 0.05 and **p < *0.01 (two-way ANOVA)* (**B** and **D**). **E** Expression levels of mRNAs in microglia isolated from JWH 133-administered WT and *App*^*NL-G-F/NL-G-F*^ mice determined using quantitative PCR. Relative expression levels of DAM marker (*Cd11c*) and complementary marker (*C1q*) are plotted as the mean ± SEM. [Veh-WT (*n* = 6), JWH 133-WT (*n* = 6), Veh-*App*^*NL-G-F/NL-G-F*^ (*n* = 6), and JWH 133- *App*^*NL-G-F/NL-G-F*^ (*n* = 6)]. *p < 0.05 and **p < *0.01 (two-way ANOVA)*. **F** Expression levels of mRNAs in astrocytes isolated from JWH 133-administered WT and *App*^*NL-G-F/NL-G-F*^ mice determined using quantitative PCR. Relative expression levels of reactive astrocytic markers (*H2d* and *Psmb8*) are plotted as means ± SEM. [Veh-WT (*n* = 5-6), JWH 133-WT (*n* = 6), Veh-*App*^*NL-G-F/NL-G-F*^ (*n* = 6), and JWH 133-*App*^*NL-G-F/NL-G-F*^ (*n* = 6)]. *p < 0.05 and **p < *0.01 (two-way ANOVA)*. **G**, **H** Representative immunofluorescence images demonstrating the expression of GFAP (green), pSTAT3 (red), or IBA1 (white) in the cerebral cortices of Veh-WT (top panels), Veh-*App*^*NL-G-F/NL-G-F*^ mice (middle panels) and JWH 133-*App*^*NL-G-F/NL-G-F*^ mice (bottom panels).　Arrowheads indicate pSTAT3- and GFAP-positive astrocytes (dashed rectangle areas have been magnified in the far right panels). Scale bars: 50 μm (magnified images) and 25 μm (others) (**G**). Percentages of pSTAT3 / GFAP cells are quantified. Values are presented as means ± SEM. [*n* = 7. Seven sections per mouse were quantified.] *p < *0.05 (Student’s t-test)* (**H**).

Gliosis with neuroinflammation is a neuropathological hallmark of the AD brain [5]. Therefore, we analyzed the expression levels of IBA1 or GFAP protein in the cerebral cortices of Veh-/JWH 133-administered WT and *App*^*NL-G-F/NL-G-F*^ mice using immunohistochemistry. Elevated expression levels of IBA1 and GFAP in microglia and astrocytes of the cerebral cortex, respectively, were attenuated by JWH 133 administration in *App*^*NL-G-F/NL-G-F*^ mice (Fig. 4A–D).

To investigate these findings in detail, we measured the mRNA expression levels of inflammatory molecules using isolated microglia and astrocytes from the cerebral cortices of Veh-/JWH 133-administered WT and *App*^*NL-G-F/NL-G-F*^ mice using RT-qPCR (Fig. 4E, F). *Cd11c* (*Itgax*), which is one of the disease associated microglia (DAM) [38] and phagocytic markers, was substantially increased in microglia isolated from JWH 133-administered *App*^*NL-G-F/NL-G-F*^ mice compared with that in Veh-administered *App*^*NL-G-F/NL-G-F*^ mice (Fig. 4E). Furthermore, the expression level of *C1q*, which is one of the inducers of the reactive astrocytes, was significantly decreased in microglia isolated from JWH 133-administered *App*^*NL-G-F/NL-G-F*^ mice (Fig. 4E). In isolated astrocytes, JWH 133-administered *App*^*NL-G-F/NL-G-F*^ mice also showed a significant decrease in reactive astrocyte markers such as *H2d* and *Psmb8*, compared with Veh-administered *App*^*NL-G-F/NL-G-F*^ mice (Fig. 4F). The recent study showed that selective deletion of the *Stat3* gene in astrocytes of APP/PS1 mice inhibited polarization of reactive astrocytes [39]. Therefore, we measured the protein level of activated / phosphorylated STAT3 in the cerebral cortex of Veh- / JWH 133-administrated *App*^*NL-G-F/NL-G-F*^ mice. The immunoreactivity of pSTAT3 was substantially decreased in JWH 133-administered *App*^*NL-G-F/NL-G-F*^ mice compared to that in Veh-administered *App*^*NL-G-F/NL-G-F*^ mice (Fig. 4G, H). These results suggest that JWH 133 ameliorates neuroinflammation in *App*^*NL-G-F/NL-G-F*^ mice through microglial CB2 receptor and suppresses polarization of reactive astrocytes by inhibiting the STAT3 pathway.

### JWH 133 administration suppresses dystrophic presynaptic terminals surrounding amyloid plaques but not amyloid deposition

**Fig. 5:**
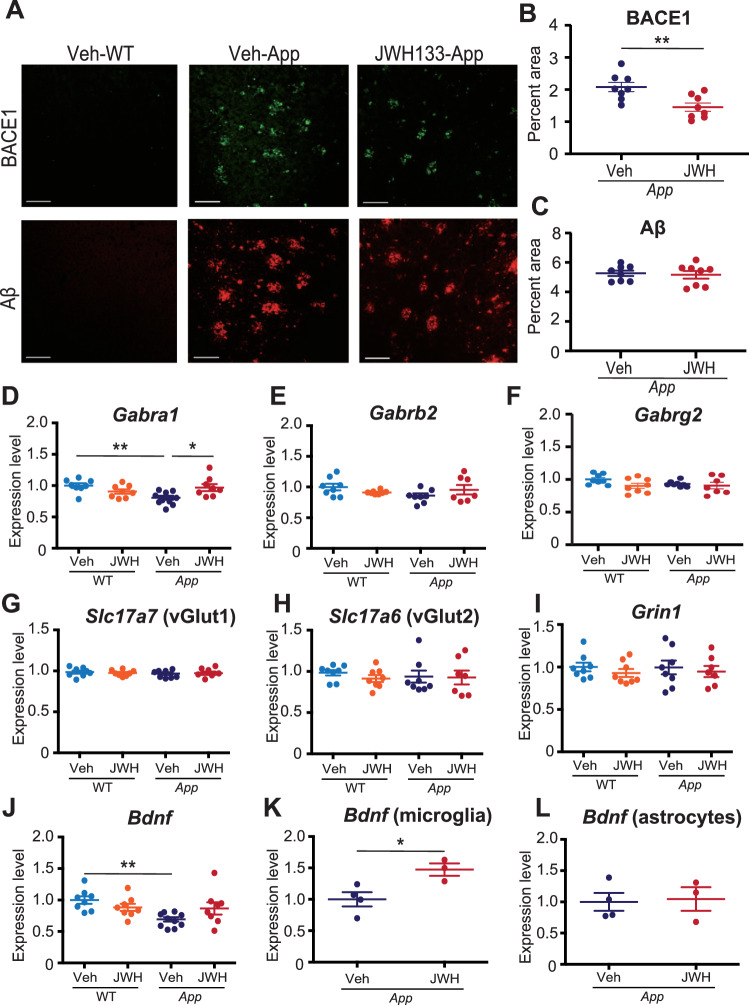
JWH 133 administration suppresses dystrophic presynaptic terminals surrounding amyloid plaques but not amyloid deposition. **A** Representative micrographs showing BACE1 (green) and Aβ (red) in the cerebral cortices of Veh- or JWH 133-administered WT or *App*^*NL-G-F/NL-G-F*^ mice. Scale bar: 100 μm. **B**, **C** Percentage of the area immunopositive for BACE1 (**B**) or Aβ (**C**) in the cerebral cortices of Veh- or JWH 133-administered *App*^*NL-G-F/NL-G-F*^ mice. Values are represented as means ± SEM. [*n* = 8. Seven sections per mouse were quantified.] *p < 0.05 and **p < *0.01 (Student’s t-test)*. **D–I** Expression levels of mRNAs in the cerebral cortices from JWH 133-administered WT and *App*^*NL-G-F/NL-G-F*^ mice were determined using quantitative PCR. Relative expression levels of GABAergic markers (*Gabra1, Gabrb2 and Gabrg2*) are plotted as means ± SEM (**D–F**). Relative expression levels of glutamatergic markers (*Slc17a7, Slc17a6 and Grin1*) are plotted as means ± SEM (**G–I**). [Veh-WT (*n* = 8), JWH 133-WT (*n* = 8), Veh-*App*^*NL-G-F/NL-G-F*^ (*n* = 8), and JWH 133-*App*^*NL-G-F/NL-G-F*^ (*n* = 7)]. *p < 0.05 and **p < 0.01 (two-way ANOVA) (**D–I**). **J–L** Expression levels of mRNAs in the cerebral cortices and glial cells isolated from JWH 133-administered WT and *App*^*NL-G-F/NL-G-F*^ mice determined using quantitative PCR. Relative expression levels of *Bdnf* are plotted as means ± SEM (**J**: cerebral cortex, **K**: microglia, and **L**: astrocytes). [Cortex: Veh-WT (*n* = 8), JWH 133-WT (*n* = 8), Veh-*App*^*NL-G-F/NL-G-F*^ (*n* = 10), and JWH 133-*App*^*NL-G-F/NL-G-F*^ (*n* = 8), microglia/astrocyte: Veh-*App*^*NL-G-F/NL-G-F*^ (*n* = 4) and JWH 133-*App*^*NL-G-F/NL-G-F*^ (*n* = 3)]. *p < 0.05 and **p < 0.01 (two-way ANOVA or *Student’s t-test*).

The presence of dystrophic neurites following amyloid accumulation has been documented in AD lesions [40]. Therefore, we investigated whether JWH 133 administration could regulate the accumulation of amyloid-β and dystrophic neurites in the cerebral cortex of *App*^*NL-G-F/NL-G-F*^ mice. We observed an increase in the expression of Bace1-immunopositive dystrophic neurites in the cerebral cortex of *App*^*NL-G-F/NL-G-F*^ mice compared with that in Veh-administered WT mice (Fig. 5A). JWH 133 administration significantly decreased the expression of dystrophic neurites in *App*^*NL-G-F/NL-G-F*^ mice (Fig. 5A, B). Aβ accumulation was increased in the brain of Veh-administered *App*^*NL-G-F/NL-G-F*^ mice compared with that in Veh-administered WT mice; however, no significant difference in Aβ levels was observed between Veh- and JWH 133-administered *App*^*NL-G-F/NL-G-F*^ mice (Fig. 5A, C).

To further investigate these findings, we examined the accumulation of soluble/insoluble Aβ in the cerebral cortex of *App*^*NL-G-F/NL-G-F*^ mice administered with JWH 133 using immunoblotting (Supplementary Fig. S2A). No differences in the levels of Tris buffer-soluble and insoluble Aβ (i.e., FA-soluble) in the cerebral cortex were found between Veh- and JWH 133- administered *App*^*NL-G-F/NL-G-F*^ mice (Supplementary Fig. S2B–E). These results suggest that microglial CB2 stimulation does not affect Aβ clearance.

Several studies have demonstrated that the excitation-inhibition (E-I) imbalance is an essential element and a critical regulator of AD pathology [41, 42]. Accordingly, we examined the mRNA expression of GABA_A_ receptor subunits in the cerebral cortex of Veh- and JWH 133- administered *App*^*NL-G-F/NL-G-F*^ mice. The expression level of α1 subunit mRNA (*Gabra1*) was significantly lower in Veh-administered *App*^*NL-G-F/NL-G-F*^ mice than in Veh-administered WT mice, and JWH 133 administration rescues its expression levels (Fig. 5D-F). No significant differences in the mRNA levels of glutamatergic transporters, such as *Slc17a7*, *Slc17a6*, and *Grin1* (Fig. 5G–I), were observed. Brain-derived neurotrophic factor (BDNF) is known to be a key molecule for cognitive function in the diseased brain [43]. Therefore, we analyzed the expression levels of *Bdnf*. We observed that the mRNA expression level of *Bdnf* was decreased in Veh-administered *App*^*NL-G-F/NL-G-F*^ mice compared with that in Veh-administered WT (Fig. 5J). Glial BDNF emerges as a critical regulator of neural activity and cognitive processes [44, 45]. To analyze the effect of JWH 133 on each glial cell type in detail, we measured the mRNA expression levels of *Bdnf* in microglia and astrocytes isolated from the cerebral cortex of Veh-/JWH 133-administered *App*^*NL-G-F/NL-G-F*^ mice using RT-qPCR. *Bdnf* was significantly increased in microglia isolated from JWH 133-administered *App*^*NL-G-F/NL-G-F*^ mice compared with that in microglia isolated from Veh-administered *App*^*NL-G-F/NL-G-F*^ mice (Fig. 5K, L). These data suggest that microglial CB2 stimulation significantly affects neuronal changes, not on the Aβ plaque deposition.

## Discussion

In this study, we report that the expression of the *CNR2/Cnr2 (CB2)* gene was commonly upregulated in the precuneus of patients with AD and in microglia isolated from the cerebral cortices of *App*^*NL-G-F/NL-G-F*^ mice using magnetic-activated cell sorting. Sustained stimulation of microglial CB2 ameliorates cognitive dysfunction in *App*^*NL-G-F/NL-G-F*^ mice by inducing beneficial neuroinflammation through controlling astrocyte activation. Furthermore, we found that JWH 133 administration ameliorated the neuronal abnormalities in *App*^*NL-G-F/NL-G-F*^ mice, such as dystrophic neurites and decreased expression of GABAergic molecules in the cerebral cortex.

CB2 is upregulated in glial cells in response to various stimuli and pathogens [46]. Some reports have shown that this response is specific to microglia [16, 47, 48], whereas others have reported CB2 expression in astrocytes [29, 49–51]. In the present study, we found that *Cnr2* was predominantly expressed in microglia and upregulated by the progression of AD pathology (Fig. 1A–C). In addition to AD mouse models, *CNR2* expression levels correlated with Aβ levels and senile plaque score in the frontal cortex of patients with AD [23]. Consistent with the cited study, we found for the first time that *CNR2* mRNA expression was upregulated in the precuneus of patients with AD (Fig. 1D, E). Studies have reported that CB2 stimulation inhibits the polarized activation of proinflammatory (M1) macrophages and microglia [11]. Conversely, one study showed that microglia derived from CB2-deficient mice inhibit the expression of proinflammatory cytokines [52]. We found that JWH 133 pretreatment suppresses the proinflammatory phenotype in primary microglia and astrocytes, likely via microglial CB2 stimulation (Fig. 2). Studies by us and others have found increased levels of *CXCL10 and TNF* in the precuneus and hippocampus of patients with AD, respectively [28, 53]. Furthermore, MHC class I (*HLA*) and *PSMB8* were known as reactive astrocytic markers, and were increased in the post-mortem human AD brains [31, 54]. Taken together, *Cnr2/CNR2* is predominantly expressed in microglia and is commonly upregulated in AD mouse models and patients with AD. Moreover, microglial CB2 stimulation may reduce the expression of proinflammatory molecules not only in the microglia but also in the astrocytes of patients with AD to control neuroinflammation.

We found that JWH 133 administration ameliorated the impairment of object recognition memory in *App*^*NL-G-F/NL-G-F*^ mice (Fig. 3B–D). This result was consistent with those of previous studies using transgenic AD mouse models such as Tg2576 and APP/PS1 mice [13, 15]. These mouse models sometimes produce artificial phenotypes, because they overproduce APP and its fragments in addition to Aβ [32]. Therefore, we performed similar experiments using *App*^*NL-G-F/NL-G-F*^ knock-in mice and concluded that CB2 stimulation is effective in ameliorating memory impairment resulting from typical amyloid pathology in *App*^*NL-G-F/NL-G-F*^ mice. Furthermore, *App*^*NL-G-F/NL-G-F*^ mice exhibited spatial memory impairment, and JWH 133 administration ameliorated the dysfunction in the Barnes maze test (Fig. 3E–G). These results provide the first evidence that CB2 stimulation is effective in improving not only object recognition memory but also spatial memory in *App*^*NL-G-F/NL-G-F*^ mice. Although CB2 is expressed at very low levels in the dopaminergic neurons and may regulate dopamine-related behavior [36, 37], JWH 133 had negligible effects on anxiety and locomotor activity in each group (Fig. 3I, J). Thus, CB2 stimulation ameliorated the cognitive impairment without neuropsychiatric side effects such as hyperlocomotion and anxiety.

Our gene expression analysis of microglia and astrocytes directly isolated from the adult mouse cerebral cortex by magnetic-activated cell sorting (MACS) provided a mechanistic view of chronic CB2 stimulation in AD mice. Previous studies have not shown changes in the expression of neuroinflammatory molecules in a cell type-specific manner. The present study demonstrated that JWH 133-administered *App*^*NL-G-F/NL-G-F*^ mice showed a significant decrease in microglial *Cd11c* and *C1q* and activated astrocyte markers, such as *H2d* and *Psmb8* (Fig. 4E, F). Cd11c is one of the DAM markers [38, 55] and is associated with phagocytosis [56]. Myeloid lineage-specific BDNF knockout mice exhibited decreased expression of CD11c^+^ cells [57]. We found that BDNF expression was increased in microglia isolated from JWH 133-administered *App*^*NL-G-F/NL-G-F*^ mice (Fig. 5K). Taken together, microglial BDNF may be associated with the upregulation of Cd11c expression. Future studies are required to clarify the role of Cd11c-positive microglia in the pathological process of AD in mice. Microglia-derived C1q is an inducer of the neurotoxic reactive astrocytes [31]. CB2 stimulation decreased microglial C1q expression, which may inhibit the induction of neurotoxic reactive astrocytes in *App*^*NL-G-F/NL-G-F*^ mice. Astrocytic STAT3 is associated with polarization of reactive astrocytes, and its deletion reduces dystrophic neurites and harmful cytokines [39]. Collectively, microglial CB2 stimulation may also prevent the induction of dystrophic neurites in *App*^*NL-G-F/NL-G-F*^ mice by reducing astrocytic pSTAT3 expression (Fig. 4G, H and Fig. 5A, B).

**Fig. 6:**
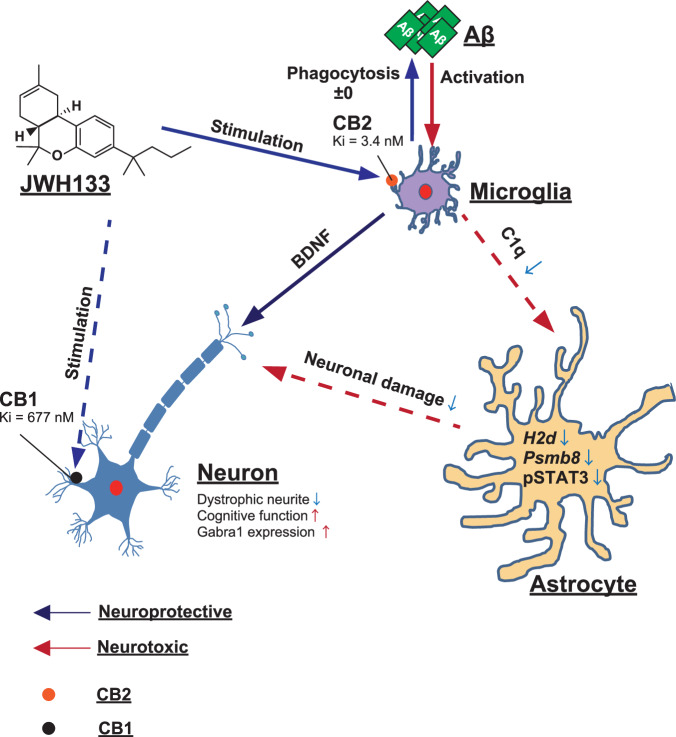
Schematic illustration of the role of microglial CB2 in ameliorating cognitive decline by regulating neuroinflammation in AD mice. JWH 133 binds to CB2 with greater affinity than CB1 and acts as a potent CB2 selective agonist. Stimulation of microglial CB2 reduces the release of C1q, which an inducer of reactive astrocytes, and induces the release of BDNF from microglia. Regulated astrocytic activation ameliorates cognitive dysfunction in *App*^*NL-G-F/NL-G-F*^ mice by protecting neuronal functions.

Moreover, we evaluated amyloid pathology and neuropathological changes in JWH 133-administered *App*^*NL-G-F/NL-G-F*^ mice. Whether CB2 stimulation is beneficial for Aβ clearance in AD model mice remains controversial [13, 15]. In this study, JWH 133 administration did not modify Aβ accumulation in the cerebral cortices of *App*^*NL-G-F/NL-G-F*^ mice (Fig. 5A, C). This result is consistent with those of a previous study [13]. According to these results, CB2 stimulation may inhibit neurotoxic inflammation and glial crosstalk, rather than directly affecting Aβ clearance (Fig. 6). Additionally, we observed a significant reduction in BACE1-immunoreactive dystrophic neurites following the administration of JWH 133 (Fig. 5A, B). The β-secretase BACE1 localizes to dystrophic presynaptic terminals surrounding Aβ plaques [58, 59]. Meanwhile, microglial BDNF was increased in JWH 133-administered *App*^*NL-G-F/NL-G-F*^ mice (Fig. 5K). A previous study showed that AM1241, a CB2 agonist, increased BDNF expression in an activated N9 microglia cell line [60]. Moreover, BDNF expression was reduced in the brains of CB2KO mice [61]. The upregulation of BDNF expression prevents neuronal cell death and mitigates synaptic dysfunction against amyloid accumulation, thereby slowing AD pathologies [62]. Based on these reports, we suggest that JWH 133 administration increased the expression of microglial BDNF, resulting in reduction in dystrophic neurites in *App*^*NL-G-F/NL-G-F*^ mice (Fig. 6).

The stability of the neural network is decreased in the AD brain, suggesting that the excitatory/inhibitory imbalance is strongly related to the pathogenesis of AD [41, 42]. In this study, we observed a decrease in the expression of *Gabra1* in the cerebral cortices of *App*^*NL-G-F/NL-G-F*^ mice, and JWH 133 increased its expression level (Fig. 5D–I). Studies have shown that the expression level of GABA type A receptors, including the α1 subunit, is significantly decreased in the entorhinal and temporal cortices of patients with AD [63]. Reactive astrocytes aberrantly release GABA, and its suppression completely restores the cognitive impairment in the mice [64]. Taken together, we hypothesize that JWH 133 administration rescues the expression of GABRA1 due to suppressed reactive astrocytes, and this promotes the amelioration of cognitive deficits (Fig. 6).

The results of the present study indicate that sustained stimulation of microglial CB2 ameliorates cognitive dysfunction in *App*^*NL-G-F/NL-G-F*^ mice by inducing beneficial neuroinflammation by controlling microglia–astrocyte crosstalk. CB2 may represent an attractive therapeutic target for treating AD and other neurodegenerative diseases involving neuroinflammation.

## Materials and methods

### Postmortem human brain tissues

Postmortem brains from 34 individuals (non-AD brain^28^ = 12, mild-AD brain^28^ = 11 and advanced AD brain = 11) were obtained by autopsy with informed consent and diagnosed by neuropathologists at the Brain Bank for Aging Research, Tokyo Metropolitan Geriatric Hospital and Institute of Gerontology. The subjects were neuropathologically grouped according to the neurofibrillary tangle staging of Braak and Braak as well as the senile plaque staging [65, 66]. The use of human postmortem brain tissue was approved by the Ethics Committees of Research Institute of Environmental Medicine, Nagoya University (approval number #328) and Tokyo Metropolitan Institute (approval number # R21-145). Brain tissue samples for RNA preparation were immediately frozen using liquid nitrogen and stored at − 80 °C before use.

### Animals

Heterozygous *App*^*+/NL-G-F*^ mice (C57BL/6-App<tm3(NL-G-F)Tcs > ), carrying the *App* gene with humanized Aβ sequence (G676R, F681Y, R684H), Swedish (KM670/671NL), Beyreuther/Iberian (I716F), and Arctic (E693G) mutations, were previously established using a knock-in strategy [32]. The homozygous *App*^*NL-G-F/NL-G-F*^ mice obtained by crossbreeding were maintained as inbred lines. Age-matched C57BL/6 Jcl mice were used as control.

All mice were maintained under a standard specific pathogen-free environment (12 h light–dark cycle; 23 ± 1 °C; 50 ± 5% humidity) with free access to food and water throughout the experiments. Animals were treated according to the guidelines of the Institutional Animal Care and Use Committee of Nagoya University (approval numbers R240025 and R240026, and #143, respectively).

### Drug administration

JWH 133 (Cayman Chemicals, MI, USA) was administered in the drinking water at a dose of 0.2 mg/kg/day using methyl acetate (0.01%) as the vehicle (Veh) for 6 months, starting at 5 months of age. The amount of water consumed by the mice was assessed every other day. The mice were randomly assigned to either Veh- or JWH 133-administration. To minimize bias because of possible undetected changes in environmental conditions, we always examined Veh-/JWH 133-administered WT/App mice in pairs; both recordings were obtained on the same day. Therefore, the experimenter was not blinded to the genotype/treatment throughout the experimental procedures. No exclusion criteria were predetermined, and no animals were excluded. The sample size for each experiment was determined based on previous studies with the relevant type of experiment [28, 67].

No difference in the body weight or ingested water was observed between the groups throughout the experiments (Supplementary Fig. S1A, B).

### Behavioral analyses

Behavioral analyses were performed at the age of 11 months. The novel object recognition test was performed, as described previously [67], except that the experiments were conducted under moderately lit conditions (12 lx).

The open field test was performed, as described previously [68] with minor modifications. The mice were placed in the center of the arena and allowed to explore the open field (40 cm × 40 cm× 30 cm) for 5 min under moderately lit conditions (100 lx). The open field was divided into an inner zone (30 cm × 30 cm), which was surrounded by an outer zone. The movement of the mice was measured using a camera mounted above the open field, and their activity was analyzed automatically using TimeOFCR1 software (O’Hara & Co., Ltd., Tokyo, Japan). Measurements included distance and time spent in the inner and outer zones.

The Barnes maze test was performed on a white circular surface (90 cm in diameter, with 12 holes equally spaced around the perimeter; Brain Science Idea, Osaka, Japan) [69]. The circular open field was elevated to a height of 75 cm above the floor. Light intensity was set at 540 lx. A black acrylic box (17 × 13 × 7 cm) was placed under one of the holes as an escape box. The location of the target hole was consistent for a given mouse, and mice within a squad were assigned to the same target hole location across sessions. We conducted each trial in a spaced fashion so that all mice within a squad completed a given trial before subsequent trials were run.

We performed training sessions for 5 days (four trials per session). The mice were individually placed in a white acrylic box (13 × 13 × 13 cm) before the start of each trial. After a 15 sec period, the white acrylic box was removed to start the trial. Each trial ended when the mouse entered the escape box or after 5 min had elapsed. If a mouse failed to find the target hole, we guided it to the hole manually and allowed it to enter the escape box and remain there for 1 min. For each trial, we measured the latency (sec) to reach the target hole using SMART (Bio Research Center, Nagoya, Japan).

One day after the last training session, the escape box was removed, and we performed a probe test for 3 min, to confirm that the mice learned the location of escape box based on navigation by distal cues in the surrounding environment. We measured the time spent in each quadrant (sec) of the visits to the target hole using the software. Hole exploration in the target quadrant was defined as the percentage of the visits to the three holes in the target quadrant for total hole visits during the test. For the reversal session, the escape box was moved to 180^o^ from its previous location, and the mice were retrained for 5 days of reversal sessions to determine the new location of the escape box. During the reversal session, we also measured the latency (sec) to reach the new target using the software.

### Microglia/astrocytes isolation from the mouse brain

MACS of microglia and astrocytes from the mouse brain was performed as described elsewhere [28]. In brief, the cerebral cortex dissected from mice transcardially perfused with phosphate-buffered saline (PBS) after deep anesthesia was dissociated at 37 °C for 15 min using the Neural Tissue Dissociation Kit-Postnatal Neurons (Miltenyi Biotec, Bergisch-Gladbach, Germany) with a gentle MACS Dissociator (Miltenyi Biotec). For microglial isolation, myelin debris was removed by using Myelin Removal Beads II (Miltenyi Biotec). Purified cells were incubated with anti-CD16/CD32 antibodies (Thermo Fisher Scientific, Waltham, MA, USA) to block Fc receptors, and then incubated with anti-CD11b microBeads (Miltenyi Biotec) to isolate microglia. CD11b-positive microglia were isolated via magnetic cell sorting through an LS column (Miltenyi Biotec). After isolating microglia, we incubated each sample with anti-ACSA2 microBeads (Miltenyi Biotec) to isolate astrocytes. ACSA2-positive astrocytes were isolated via magnetic cell sorting through an LS column (Miltenyi Biotec).

### Quantification of mRNA levels by real-time PCR

Total RNA was extracted from MACS-sorted microglia using the RNeasy Micro Kit (Qiagen) as described previously [28]. Complementary DNA (cDNA) from MACS sorted cells was generated and amplified from 2.5 or 5 ng of total RNA by using the PrimeScript™ RT Reagent Kit (Perfect Real Time) (TaKaRa Bio, Kusatsu, Japan), and 1/50 of the yield was amplified with the SYBR Premix Ex Taq II (Tli RNaseH Plus) (TaKaRa Bio) using the Thermal Cycler Dice Real Time System II or III (TaKaRa Bio). The thermocycle protocol was as follows: 1 cycle at 95 °C for 30 sec, 40 cycles at 95 °C for 5 sec and 60°C for 30 sec, and a dissociation stage at 95 °C for 15 sec, 60 °C for 30 sec, and 95 °C for 15 sec. Actin was used for normalization. The primers used are listed in Supplementary Table S1.

### Immunofluorescence study

Immunofluorescence analysis was performed as described previously [67]. In brief, the mice were deeply anesthetized and perfused intracardially with 4% paraformaldehyde in PBS. The brains were dissected, post-fixed with the same fixative, and cryoprotected with 30% sucrose in PBS. Twenty-micrometer-thick coronal brain sections were fixed with 4% paraformaldehyde in PBS for 5 min and permeabilized with 0.1% Triton X-100/PBS for 10 min. After an incubation in blocking solution (5% goat or donkey serum/PBS) for 1 h, the sections were incubated with a combination of the following antibodies: rabbit anti-Iba-1 (#019–19741, 1:500; FUJIFILM Wako, Osaka, Japan), goat anti-AIF1/Iba-1 (#NB100-1028, 1:250; Novus Biologicals, Littleton, CO, USA), mouse anti-GFAP (#G3893, 1:500; Merck KGaA, Darmstadt, Germany), rabbit anti-BACE1 (#5606, 1:200, Cell Signaling Technology, Danvers, MA, USA), rabbit anti-phospho-STAT3 (Tyr705) (#9145, 1:200, Cell Signaling Technology), or mouse anti-Human Amyloid-β (N) (82E1) (#10323, 1:200; Immuno-Biological Laboratories Co., Ltd., Gunma, Japan) at 4 °C overnight. After washing with PBS, the sections were incubated with fluorescent-conjugated anti-rabbit, anti-mouse, or anti-goat IgGs (1:1,000; Thermo Fisher Scientific) or Streptavidin, Alexa Fluor™ 546 conjugate (1:500; Thermo Fisher Scientific) at room temperature for 1 h. After washing in PBS, the sections were mounted on slides and analyzed using a confocal microscope (LSM700, Carl Zeiss, Oberkochen, Germany). Quantitative analysis of microscopic images was performed using ImageJ (National Institutes of Health).

### Protein extraction and Western blotting

Protein extraction was performed as previously described [70]. In brief, mouse brains were homogenized in 5× volumes of Tris buffer (50 mM Tris–HCl pH7.6, 150 mM NaCl, complete protease inhibitor cocktail, and PhosSTOP phosphatase inhibitor cocktail) with 25 strokes using a potter homogenizer and centrifuged at 200,000 × g for 20 min at 4 °C. The resultant supernatant was collected as a brain Tris buffer-soluble (TBS) fraction. After the addition of the same amount of 2% Triton X-100/Tris buffer, the pellet was homogenized on ice and centrifuged at 200,000 × g for 20 min at 4°C. The resultant supernatant was collected as the brain Triton X fraction. Then, the same amount of 2% SDS containing Tris buffer was added to the pellet. After homogenization at room temperature, the pellet was incubated for 2 h at 37 °C and centrifuged at 200,000 × g for 20 min at 20 °C. The resultant supernatant was collected as the brain SDS fraction. Finally, the pellet was sonicated with 500 μL of 70% formic acid (WAKO) solution. The samples were centrifuged at 200,000 × g for 20 min at 4 °C, and the resultant supernatant was evaporated for 2 h. The pellet was dissolved in the same volume of DMSO as the brain weight and stored at −80 °C until use as the formic acid-soluble (FA) fraction. The BCA assay was performed to estimate the protein concentration. Equal amounts of total protein were separated via sodium dodecyl sulfate-polyacrylamide electrophoresis (SDS-PAGE) and transferred to a polyvinylidene difluoride membrane (Immobilon-P; Merck Millipore, Billerica, MA, USA). The membrane was incubated with a blocking buffer (50 mM Tris-HCl (pH7.4), 150 mM NaCl, 0.05% (v/v) Tween-20, and 2% (w/v) bovine serum albumin (FUJIFILM Wako), followed by incubation with mouse anti-Aβ (#10323, 1:200; IBL, Gunma, Japan) or mouse anti-ACTB (#a5441,1:5000; Sigma–Aldrich, St. Louis, MO) antibody diluted in the blocking buffer at 4 °C for at least 6 h. Bound antibodies were detected using horseradish peroxidase-conjugated secondary antibodies and Immobilon Crescendo Western HRP substrate (Merck Millipore). Images were obtained using LAS-4000 mini (Cytiva, Shinjuku, Tokyo, Japan) with the equipped software (Multi-Gauge; Cytiva).

The gels were stained with SYPRO Ruby Protein Stains (BioRad, Hercules, CA, USA) according to the manufacturer’s protocols. Images were acquired using FAS-IV (Nippon Genetics, Tokyo, Japan).

### Glial culture and drug treatment

Microglial cultures were prepared as described previously [71] with minor modifications. In brief, brains from mice at postnatal days 1-2 were dissociated in 0.25% Trypsin and 10 mg/mL Dnase I containing PBS at 37 °C for 10 min. Dissociated cells were washed and plated on poly-L-lysine-coated flasks in 10% FBS DMEM (DMEM medium supplemented with 10% FBS, 50 U/mL penicillin, and 50 µg /mL streptomycin) (Life Technologies) in a 5% CO_2_ incubator. One to two weeks after seeding the cells dissociated from neonatal mouse brains, microglia began to appear on the top of the astrocytic monolayer. Microglia were separated from the underlying astrocytic monolayer, seeded on culture dishes, and ready for use in experiments. The resulting astrocyte layer was detached using 0.25% trypsin and replated onto culture plates. After replating twice, the cultured astrocytes were used as primary astrocytes in all experiments and maintained in 10% FBS DMEM in a 5% CO_2_ incubator.　Microglia were pretreated with 5 µM JWH 133 for 1 h and then stimulated with IFN-γ (10 ng/mL; Peprotech EC Ltd, London, UK) for 6 h. RNA samples were isolated from microglia 6 h after IFN-γ stimulation.

Microglia-conditioned medium (MCM) was prepared as described previously [31] with minor modifications. Briefly, primary microglia were pretreated with 5 μM JWH 133 or DMSO (vehicle; Veh) for 1 h followed by treatment with lipopolysaccharide (LPS) (50 ng/mL; Sigma) for 24 h, and the medium was then used as MCM. The culture medium of primary astrocytes was replaced with Veh / LPS- or JWH 133 / LPS-MCM 24 h after seeding. RNA samples were isolated from astrocytes 24 h after MCM treatment. RT-qPCR methods are described above.

### Statistical analysis

All data are expressed as means ± SEM. We did not assess the normality of the data before the statistical comparisons. We opted to use parametric statistics for consistency across experiments and provided evidence that analysis of variance (ANOVA) is robust to slight non-normality [72, 73]. No test for outliers was performed. One-way, two-way, or three-way with or without repeated measures ANOVA was used, followed by Tukey’s test when F ratios were significant (*p* < 0.05). Significant differences between two groups were assessed using the Student’s t-test. All analyses were performed using GraphPad Prism software (GraphPad Software, La Jolla, CA, USA). Detailed information on the statistical analysis is summarized in Supplementary Table S2.

## Supplementary information

Supplementary Figure S1-2

Supplementary Table S1

Supplementary Table S2

Unedited blot and gel images

## Data Availability

Data, material, and software information supporting the conclusions of this article is included within the article and its Supplementary information. Data will be made available on request.
