# Supplementary material for: Microglial cannabinoid receptor type II stimulation improves cognitive impairment and neuroinflammation in Alzheimer’s disease mice by controlling astrocyte activation
Source: Cell Death Dis. 2024 Nov 26;15(11):858. doi: 10.1038/s41419-024-07249-6 (PMC11589152; doi:10.1038/s41419-024-07249-6)
Supplement: Supplementary file 1 — Supplementary Figure S1-2 [file 41419_2024_7249_MOESM1_ESM.pdf]

Supplementary figures

Microglial cannabinoid receptor type II stimulation improves cognitive impairment and neuroinflammation in Alzheimer's disease model mice by controlling astrocyte activation

Akira Sobue, Okiru Komine, Fumito Endo, Chihiro Kakimi, Yuka Miyoshi, Noe Kawade, Seiji Watanabe, Yuko Saito, Shigeo Murayama, Takaomi C Saido, Takashi Saito, Koji Yamanaka

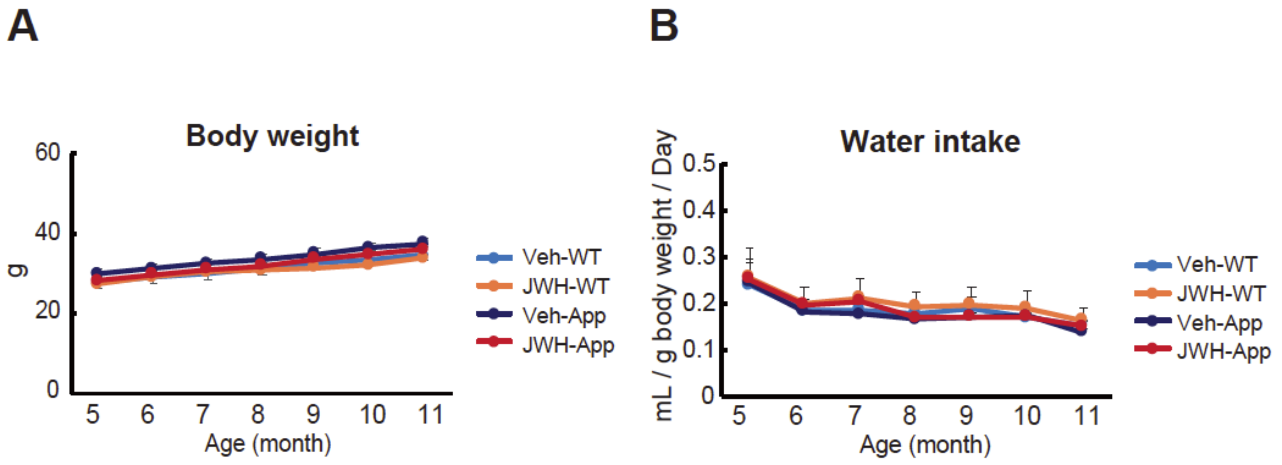

Figure S1. No difference in the body weight or the water intake between the groups throughout the experiment.

A Body weight of Veh- / JWH 133-administered WT and *App*$^{NL\text{-}G\text{-}F/NL\text{-}G\text{-}F}$ mice [Veh-WT (n = 18), JWH 133-WT (n = 15), Veh-*App*$^{NL\text{-}G\text{-}F/NL\text{-}G\text{-}F}$ (n = 21), and JWH 133-*App*$^{NL\text{-}G\text{-}F/NL\text{-}G\text{-}F}$ (n = 19)]. Values are presented as means ± SEM (*repeated measures three-way ANOVA*).

B Water intake of WT and *App*$^{NL\text{-}G\text{-}F/NL\text{-}G\text{-}F}$ mice [Veh-WT (n = 6 cages; 18 mice), JWH 133-WT (n = 5 cages; 14 mice), Veh-*App*$^{NL\text{-}G\text{-}F/NL\text{-}G\text{-}F}$ (n = 6 cages; 21 mice), and JWH 133-*App*$^{NL\text{-}G\text{-}F/NL\text{-}G\text{-}F}$ (n = 7 cages; 19 mice)]. Values are presented as means ± SEM. (*repeated measures three-way ANOVA*).

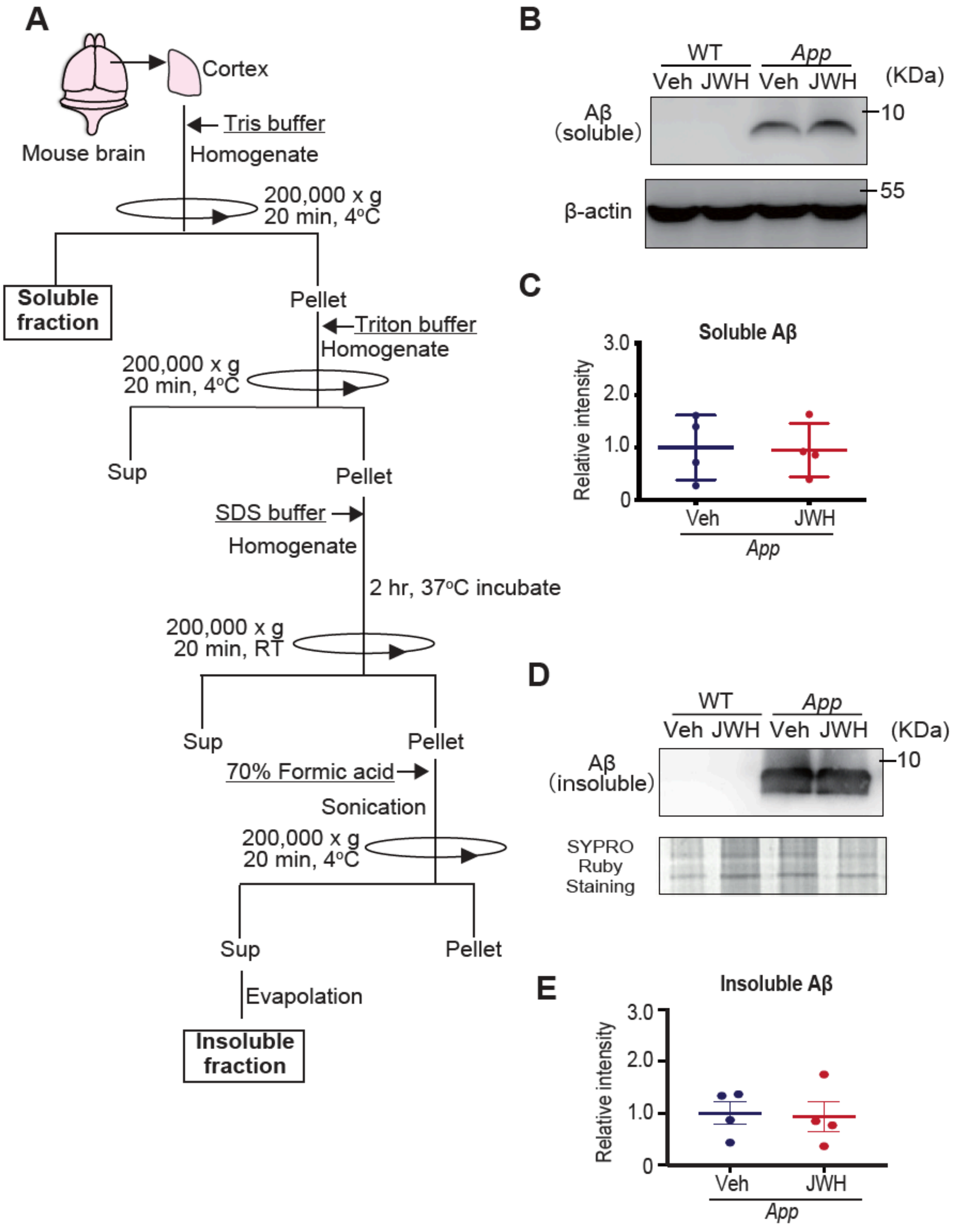

Figure S2. The chronic administration of JWH 133 marginally affected the Aβ levels in the cerebral cortices of *App*$^{NL\text{-}G\text{-}F/NL\text{-}G\text{-}F}$ mice.

A A schematic protocol for protein extraction from the cerebral cortices of Veh / JWH 133-administered WT and *App*$^{NL\text{-}G\text{-}F/NL\text{-}G\text{-}F}$ mice.

B Representative immunoblotting images for Aβ and β-Actin levels in Tris buffer-soluble (TBS) fractions extracted from the cerebral cortices of Veh- / JWH 133-administered WT and *App*$^{NL\text{-}G\text{-}F/NL\text{-}G\text{-}F}$ mice at 12 months old.

C Relative protein levels of Aβ in the TBS fraction (in **B**). Values are presented as means ± SEM [Veh-*App*$^{NL\text{-}G\text{-}F/NL\text{-}G\text{-}F}$ (n = 4) and JWH 133-*App*$^{NL\text{-}G\text{-}F/NL\text{-}G\text{-}F}$ (n = 4)]. (*Student's t-test*).

D A representative immunoblotting image for Aβ level and SYPRO Ruby staining in FA fractions extracted from the cerebral cortices of Veh / JWH 133-administered WT and *App*$^{NL\text{-}G\text{-}F/NL\text{-}G\text{-}F}$ mice at 12 months old.

E Relative protein levels of Aβ in the FA fraction (in **D**). Values are presented as means + SEM [Veh-*App*$^{NL\text{-}G\text{-}F/NL\text{-}G\text{-}F}$ (n = 4) and JWH 133-*App*$^{NL\text{-}G\text{-}F/NL\text{-}G\text{-}F}$ (n = 4)]. (*Student's t-test*).
