# Supplementary material for: Microglial cannabinoid receptor type II stimulation improves cognitive impairment and neuroinflammation in Alzheimer’s disease mice by controlling astrocyte activation
Source: Cell Death Dis. 2024 Nov 26;15(11):858. doi: 10.1038/s41419-024-07249-6 (PMC11589152; doi:10.1038/s41419-024-07249-6)
Supplement: Supplementary file 2 — Supplementary Table S1 [file 41419_2024_7249_MOESM2_ESM.docx]

**Table S1. The primer sequences used for quantitative RT-PCR**
