# Supplementary material for: Microglial cannabinoid receptor type II stimulation improves cognitive impairment and neuroinflammation in Alzheimer’s disease mice by controlling astrocyte activation
Source: Cell Death Dis. 2024 Nov 26;15(11):858. doi: 10.1038/s41419-024-07249-6 (PMC11589152; doi:10.1038/s41419-024-07249-6)
Supplement: Supplementary file 3 — Supplementary Table S2 [file 41419_2024_7249_MOESM3_ESM.pdf]

Table S2. Summary of statistical analysis

Figure	Molecule / parameter	Number of samples	Test used	Degree of freedom and F/t/p value	Post-hoc test	Significance
1B	*Cnr2*	WT: n = 4,<br>*App-KI*: n = 4	Likelihood ratio test	-	-	WT vs. *App-KI*: *q = 1.73E-09*
1C	*Microglial Cnr2*	WT (2M): n = 4,<br>*App-KI* (2M): n = 4<br>WT (4M): n = 4,<br>*App-KI* (4M): n = 4<br>WT (8M): n = 4,<br>*App-KI* (8M): n = 4	Two-way ANOVA	*Genotype, F (1, 18) = 16.61, p = 0.0007,*<br>*Age, F (2, 18) = 5.694, p = 0.0121,*<br>*Activation x Treatment, F (2, 18) = 5.684, p = 0.0122*	Tukey's multiple comparisons test	8M WT vs. 8M *App-KI*: *p = 0.0009*<br>*2M App-KI vs. 8M App-KI: p = 0.0058*<br>*4M App-KI vs. 8M App-KI: p = 0.0085*
	Astrocytic Cnr2	WT (2M): n = 4,<br>*App-KI* (2M): n = 4<br>WT (4M): n = 4,<br>*App-KI* (4M): n = 4<br>WT (8M): n = 4,<br>*App-KI* (8M): n = 4	Two-way ANOVA	*Genotype, F (1, 18) = 4.475, p = 0.0486,*<br>*Age, F (2, 18) = 1.934, p = 0.1735,*<br>*Activation x Treatment, F (2, 18) = 1.999, p = 0.1644*	Tukey's multiple comparisons test	N.A.
1E	*CNR2*	non-AD: n = 12,<br>Mild AD: n =11,<br>Advanced AD: n = 11	One-way ANOVA	*F (2, 31) = 6.068, p = 0.0060*	Tukey's multiple comparisons test	non-AD vs. Advanced AD: *p = 0.0375,*<br>Mild AD vs. Advanced AD: *p = 0.0063*
2B	*Tnf*	Veh-PBS: n = 6,<br>JWH 133-PBS: n = 6,<br>Veh-IFNγ: n = 6,<br>JWH 133-IFNγ: n = 6	Two-way ANOVA	*Activation, F (1, 20) = 98.65, p < 0.0001,*<br>*Treatment, F (1, 20) = 5.647, p = 0.0276,*<br>*Activation x Treatment, F (1, 20) = 3.072, p = 0.0950*	Tukey's multiple comparisons test	Veh-PBS vs. Veh-IFNγ: *p < 0.0001* ,<br>Veh-IFNγ vs. JWH 133-IFNγ: *p = 0.0390*
2C	*Cxcl10*		Two-way ANOVA	*Activation, F (1, 20) = 173.5, p < 0.0001,*<br>*Treatment, F (1, 20) = 3.931, p = 0.0613,*<br>*Activation x Treatment, F (1, 20) = 4.150, p = 0.0551*	Tukey's multiple comparisons test	Veh-PBS vs. Veh-IFNγ: *p < 0.0001* ,<br>Veh-IFNγ vs. JWH 133-IFNγ: *p = 0.0457*
2E	*Psmb8*	Veh-PBS MCM: n = 6,<br>JWH 133-PBS MCM: n = 6,<br>PBS-LPS MCM: n = 6,<br>JWH 133-LPS MCM: n = 6	Two-way ANOVA	*Activation, F (1, 20) = 1275, p < 0.0001,*<br>*Treatment, F (1, 20) = 13.98, p = 0.0013,*<br>*Activation x Treatment, F (1, 20) = 12.14, p = 0.0023*	Tukey's multiple comparisons test	Veh-PBS MCM vs. Veh-LPS MCM: *p < 0.0001* ,<br>Veh-LPS MCM vs. JWH 133-LPS MCM: *p = 0.0003*
2F	*H2d*		Two-way ANOVA	*Activation, F (1, 20) = 98.44, p < 0.0001,*<br>*Treatment, F (1, 20) = 4.647, p = 0.0435,*<br>*Activation x Treatment, F (1, 20) = 3.957, p = 0.0605*	Tukey's multiple comparisons test	Veh-PBS MCM vs. Veh-LPS MCM: *p < 0.0001* ,<br>Veh-LPS MCM vs. JWH 133-LPS MCM: *p = 0.0381*
3C, D	Exploratory preference (Training)	Veh-WT: n = 9,<br>JWH 133-WT: n = 8,<br>Veh-*App-KI*: n = 9,<br>JWH 133-*App-KI*: n = 9	Two-way ANOVA	*Genotype: F (1, 30) = 4.183, p = 0.0497,*<br>*Administration: F (1, 30) = 1.546, p = 0.2233,*<br>*Genotype × Administration: F (1, 30) = 0.00287, p = 0.9577*	Tukey's multiple comparisons test	N.A.
	Exploratory preference (Test)		Two-way ANOVA	*Genotype: F (1, 30) = 16.49, p = 0.0003,*<br>*Administration: F (1, 30) = 15.96, p = 0.0004,*<br>*Genotype × Administration: F (1, 30) = 4.754, p = 0.0372*	Tukey's multiple comparisons test	Veh-WT vs. Veh-*App-KI*: *p = 0.0007* ,<br>Veh-*App-KI* vs. JWH 133-*App-KI*: *p = 0.0008*
	Exploratory time (Training)		Two-way ANOVA	*Genotype: F (1, 30) = 2.224, p = 0.1463,*<br>*Administration: F (1, 30) = 0.8526, p = 0.3632,*<br>*Genotype × Administration: F (1, 30) = 0.2508, p = 0.6202*	Tukey's multiple comparisons test	N.A.
	Exploratory time (Test)		Two-way ANOVA	*Genotype: F (1, 30) = 0.2122, p = 0.6484,*<br>*Administration: F (1, 30) = 0.2599, p = 0.6139,*<br>*Genotype × Administration: F (1, 30) = 0.7261, p = 0.4009*	Tukey's multiple comparisons test	N.A.
3F	Training	Veh-WT: n = 9,<br>JWH 133-WT: n = 8,<br>Veh-*App-KI*: n = 15,<br>JWH 133-*App-KI*: n = 10	Repeated measure three-way ANOVA	*Days: F (2.489, 103.3) = 107.4, p < 0.0001,*<br>*Genotype: F (1, 42) = 2.381, p = 0.1303,*<br>*Administration: F (1, 42) = 0.1802, p = 0.6733,*<br>*Days x Genotype: F (4, 166) = 0.1543, p = 0.9608,*<br>*Days x Administration: F (4, 166) = 0.5237, p = 0.7184,*<br>*Genotype x Administration: F (1, 42) = 0.1946, p = 0.6614,*<br>*Days x Genotype x Administration: F (4, 166) = 0.9206, p = 0.4534*	Tukey's multiple comparisons test	N.A.
	Reveasal		Two-way ANOVA	*Genotype: F (1, 42) = 0.01665, p = 0.8979,*<br>*Administration: F (1, 42) = 1.520, p = 0.2244,*<br>*Genotype × Administration: F (1, 42) = 0.04974, p = 0.8246*	Tukey's multiple comparisons test	N.A.
3G	Target	Veh-WT: n = 9,<br>JWH 133-WT: n = 8,<br>Veh-*App-KI*: n = 15,<br>JWH 133-*App-KI*: n = 10	Two-way ANOVA	*Genotype: F (1, 42) = 12.65, p = 0.0009,*<br>*Administration: F (1, 42) = 0.8060, p = 0.3744,*<br>*Genotype × Administration: F (1, 42) = 13.74, p = 0.0006*	Tukey's multiple comparisons test	Veh-WT vs. Veh-*App-KI*: *p < 0.0001,*<br>Veh-*App-KI* vs. JWH 133-*App-KI*: *p = 0.0041*
	Left		Two-way ANOVA	*Genotype: F (1, 42) = 2.719, p = 0.1066,*<br>*Administration: F (1, 42) = 0.05484, p = 0.8160,*<br>*Genotype × Administration: F (1, 42) = 0.2641, p = 0.6100*	Tukey's multiple comparisons test	N.A.
	Right		Two-way ANOVA	*Genotype: F (1, 42) = 1.170, p = 0.2855,*<br>*Administration: F (1, 42) = 0.1571, p = 0.6938,*<br>*Genotype × Administration: F (1, 42) = 0.9107, p = 0.3454*	Tukey's multiple comparisons test	N.A.
	Oppsite		Two-way ANOVA	*Genotype: F (1, 42) = 1.809, p = 0.1858,*<br>*Administration: F (1, 42) = 1.726, p = 0.1960,*<br>*Genotype × Administration: F (1, 42) = 7.536, p = 0.0089*	Tukey's multiple comparisons test	Veh-WT vs. Veh-*App-KI*: *p = 0.0198*
3I	Outer zone	Veh-WT: n = 8,<br>JWH 133-WT: n = 7,<br>Veh-*App-KI*: n = 13,<br>JWH 133-*App-KI*: n = 12	Two-way ANOVA	*Genotype: F (1, 36) = 0.2629, p = 0.6113,*<br>*Administration: F (1, 36) = 0.1722, p = 0.6807,*<br>*Genotype × Administration: F (1, 36) = 0.9732, p = 0.3305*	Tukey's multiple comparisons test	*N.A.*
	Inner zone		Two-way ANOVA	*Genotype: F (1, 36) = 0.2584, p = 0.6143,*<br>*Administration: F (1, 36) = 0.1685, p = 0.6839,*<br>*Genotype × Administration: F (1, 36) = 0.9651, p = 0.3325*	Tukey's multiple comparisons test	*N.A.*
3J	Total distance		Two-way ANOVA	*Genotype: F (1, 36) = 1.167, p = 0.2872,*<br>*Administration: F (1, 36) = 0.2391, p = 0.6278,*<br>*Genotype × Administration: F (1, 36) = 0.1181, p = 0.7331*	Tukey's multiple comparisons test	*N.A.*
4B	IBA1	Veh-WT: n = 8 (56 sections),<br>JWH 133-WT: n = 8 (56 sections),<br>Veh-*App-KI*: n = 8 (56 sections),<br>JWH 133-*App-KI*: n = 8 (56 sections)	Two-way ANOVA	*Genotype: F (1, 28) = 71.87, p < 0.0001,*<br>*Administrationt: F (1, 28) = 15.44, p = 0.0005,*<br>*Genotype × Administration: F (1, 28) = 15.26, p = 0.0005*	Tukey's multiple comparisons test	Veh-WT vs. Veh-*App-KI*: *p < 0.0001,*<br>Veh-*App-KI* vs. JWH 133-*App-KI*: *p < 0.0001*
4D	GFAP	Veh-WT: n = 7 (49 sections),<br>JWH 133-WT: n = 7 (49 sections),<br>Veh-*App-KI*: n = 7 (49 sections),<br>JWH 133-*App-KI*: n = 7 (49 sections)	Two-way ANOVA	*Genotype: F (1, 24) = 541.0, p < 0.0001,*<br>*Administrationt: F (1, 24) = 39.50, p < 0.0001,*<br>*Genotype × Administration: F (1, 24) = 37.55, p < 0.0001*	Tukey's multiple comparisons test	Veh-WT vs. Veh-*App-KI*: *p < 0.0001,*<br>Veh-*App-KI* vs. JWH 133-*App-KI*: *p < 0.0001*
4E	*Cd11c*	Veh-WT: n = 6,<br>JWH 133-WT: n = 6,<br>Veh-*App-KI*: n = 6,<br>JWH 133-*App-KI*: n = 6	Two-way ANOVA	*Genotype: F (1, 20) = 314.2, p < 0.0001,*<br>*Administration: F (1, 20) = 4.905, p = 0.0386,*<br>*Genotype × Administration: F (1, 20) = 4.852, p = 0.0395*	Tukey's multiple comparisons test	Veh-WT vs. Veh-*App-KI*: *p < 0.0001,*<br>Veh-*App-KI* vs. JWH 133-*App-KI*: *p = 0.0253*
	C1q	Veh-WT: n = 6,<br>JWH 133-WT: n = 6,<br>Veh-*App-KI*: n = 6,<br>JWH 133-*App-KI*: n = 6	Two-way ANOVA	*Genotype: F (1, 20) = 184.0, p < 0.0001,*<br>*Administration: F (1, 20) = 11.51, p = 0.0029,*<br>*Genotype × Administration: F (1, 23) = 9.711, p = 0.0054*	Tukey's multiple comparisons test	Veh-WT vs. Veh-*App-KI*: *p < 0.0001,*<br>Veh-*App-KI* vs. JWH 133-*App-KI*: *p = 0.0009*
4F	*H-2d*	Veh-WT: n = 6,<br>JWH 133-WT: n = 6,<br>Veh-*App-KI*: n = 6,<br>JWH 133-*App-KI*: n = 6	Two-way ANOVA	*Genotype: F (1, 20) = 113.1, p < 0.0001,*<br>*Administration: F (1, 20) = 5.850, p = 0.0252,*<br>*Genotype × Administration: F (1, 20) = 3.355, p = 0.0819*	Tukey's multiple comparisons test	Veh-WT vs. Veh-*App-KI*: *p < 0.0001,*<br>Veh-*App-KI* vs. JWH 133-*App-KI*: *p = 0.0325*
	Psmb8	Veh-WT: n = 5,<br>JWH 133-WT: n = 6,<br>Veh-*App-KI*: n = 6,<br>JWH 133-*App-KI*: n = 6	Two-way ANOVA	*Genotype: F (1, 19) = 64.78, p < 0.0001,*<br>*Administration: F (1, 19) = 4.338, p = 0.0510,*<br>*Genotype × Administration: F (1, 19) = 4.221, p = 0.0539*	Tukey's multiple comparisons test	Veh-WT vs. Veh-*App-KI*: *p < 0.0001,*<br>Veh-*App-KI* vs. JWH 133-*App-KI*: *p = 0.0342*
4H	pSTAT3	Veh-*App-KI*: n = 7 (49 sections),<br>JWH 133-*App-KI*: n = 7 (49 sections)	Student t-test	*t = 11.66, df = 12*	-	Veh-*App-KI* vs. JWH 133-*App-KI*: *p < 0.0001*
5B	BACE1	Veh-*App-KI*: n = 8 (56 sections),<br>JWH 133-*App-KI*: n = 8 (56 sections)	Student t-test	*t = 3.250, df = 14*	-	Veh-*App-KI* vs. JWH 133-*App-KI*: *p = 0.0058*
5C	Aβ		Student t-test	*t = 0.3209, df = 14*	-	Veh-*App-KI* vs. JWH 133-*App-KI*: *p = 0.7531*
5D	*Gabra1*	Veh-WT: n = 8,<br>JWH 133-WT: n = 8,<br>Veh-*App-KI*: n = 10,<br>JWH 133-*App-KI*: n = 8	Two-way ANOVA	*Genotype: F (1, 30) = 2.625, p = 0.1157,*<br>*Administration: F (1, 30) = 0.7085, p = 0.4066,*<br>*Genotype × Administration: F (1, 30) = 10.12, p = 0.0034*	Tukey's multiple comparisons test	Veh-WT vs. Veh-*App-KI*: *p = 0.0079* ,<br>Veh-*App-KI* vs. JWH 133-*App-KI*: *p = 0.0314*
5E	*Gabrb2*	Veh-WT: n = 8,<br>JWH 133-WT: n = 8,<br>Veh-*App-KI*: n = 8,<br>JWH 133-*App-KI*: n = 7	Two-way ANOVA	*Genotype: F (1, 30) = 1.871, p = 0.1815,*<br>*Administration: F (1, 30) = 0.05343, p = 0.8188,*<br>*Genotype × Administration: F (1, 30) = 4.819, p = 0.0360*	Tukey's multiple comparisons test	*N.A.*
5F	*Gabrg2*		Two-way ANOVA	*Genotype: F (1, 27) = 3.106, p = 0.0893,*<br>*Administration: F (1, 27) = 0.8698, p = 0.3593,*<br>*Genotype × Administration: F (1, 27) = 1.108, p = 0.3018*	Tukey's multiple comparisons test	*N.A.*
5G	*Slc17a7*<br>*(vGlut1)*	Veh-WT: n = 8,<br>JWH 133-WT: n = 8,<br>Veh-*App-KI*: n = 8,<br>JWH 133-*App-KI*: n = 7	Two-way ANOVA	*Genotype: F (1, 27) = 0.3532, p = 0.5572,*<br>*Administration: F (1, 27) = 0.05964, p = 0.8089,*<br>*Genotype × Administration: F (1, 27) = 0.1659, p = 0.6870*	Tukey's multiple comparisons test	*N.A.*
5H	*Slc17a6*<br>*(vGlut2)*		Two-way ANOVA	*Genotype: F (1, 27) = 0.07694, p = 0.7836,*<br>*Administration: F (1, 27) = 0.4575, p = 0.5046,*<br>*Genotype × Administration: F (1, 27) = 0.2330, p = 0.6332*	Tukey's multiple comparisons test	*N.A.*
5I	*Grin1*		Two-way ANOVA	*Genotype: F (1, 27) = 0.01215, p = 0.9130,*<br>*Administration: F (1, 27) = 0.9415, p = 0.3405,*<br>*Genotype × Administration: F (1, 27) = 0.02503, p = 0.8755*	Tukey's multiple comparisons test	*N.A.*
5J	*Bdnf*	Veh-WT: n = 8,<br>JWH 133-WT: n = 8,<br>Veh-*App-KI*: n = 10,<br>JWH 133-*App-KI*: n = 8	Two-way ANOVA	*Genotype: F (1, 30) = 6.568, p = 0.0156,*<br>*Administration: F (1, 30) = 0.2002, p = 0.6578,*<br>*Genotype × Administration: F (1, 30) = 5.250, p = 0.0291*	Tukey's multiple comparisons test	Veh-WT vs. Veh-*App-KI*: *p = 0.0071*
5K	*Microglial Bdnf*	Veh-*App-KI*: n = 4,<br>JWH 133-*App-KI*: n = 3	Student t-test	*t = 2.424, df = 5*	-	Veh-*App-KI* vs. JWH 133-*App-KI*: *p = 0.0293*
5L	*Astrocytic Bdnf*		Student t-test	*t = 2.424, df = 5*	-	*N.A.*

Figure	Molecule / parameter	Number of samples	Test used	Degree of freedom and F/t/p value	Post-hoc test	Significance
S1A	Body weight	Veh-WT: n = 18,<br>JWH 133-WT: n = 14,<br>Veh-*App-KI*: n = 21,<br>JWH 133-*App-KI*: n = 19	Repeated measure three-way ANOVA	*Months: F (1.4, 96.63) = 189.4, p < 0.0001,*<br>*Genotype: F (1, 69) = 1.026, p = 1.026,*<br>*Administration: F (1, 69) = 3.269, p = 0.0749,*<br>*Months x Genotype: F (6, 414) = 1.543, p = 0.941,*<br>*Month x Administration: F (6, 414) = 2.172, p = 0.0447,*<br>*Genotype x Administration: F (1, 69) = 0.1834, p = 0.6698,*<br>*Month x Genotype x Administration: F (6, 414) = 0.8330, p = 0.5448*	Tukey's multiple comparisons test	*N.A.*
S1B	Water intake	Veh-WT: n = 6 cages (18 mice),<br>JWH 133-WT: n = 6 cages (14 mice),<br>Veh-*App-KI*: n = 5 cages (21 mice),<br>JWH 133-*App-KI*: n = 7 cages (19 mice)	Repeated measure three-way ANOVA	*Months: F (1.494, 29.87) = 9.952, p = 0.0013,*<br>*Genotype: F (1, 20) = 0.4586, p = 0.5060,*<br>*Administration: F (1, 20) = 0.3117, p = 0.5829,*<br>*Months x Genotype: F (6, 120) = 0.1485, p = 0.989,*<br>*Month x Administration: F (6, 120) = 0.2037, p = 0.9751,*<br>*Genotype x Administration: F (1, 69) = 0.03812, p = 0.8472,*<br>*Month x Genotype x Administration: F (6, 120) = 0.0507, p = 0.9995*	Tukey's multiple comparisons test	*N.A.*
S2C	Soluble Aβ	Veh-*App-KI*: n = 4,<br>JWH 133-*App-KI*: n = 4	Student t-test	*t = 0.1168, df = 6*	-	*N.A.*
S2E	Insoluble Aβ	Veh-*App-KI*: n = 4,<br>JWH 133-*App-KI*: n = 4	Student t-test	*t = 0.9978, df = 6*	-	*N.A.*
