# Supplementary material for: Microglial cannabinoid receptor type II stimulation improves cognitive impairment and neuroinflammation in Alzheimer’s disease mice by controlling astrocyte activation
Source: Cell Death Dis. 2024 Nov 26;15(11):858. doi: 10.1038/s41419-024-07249-6 (PMC11589152; doi:10.1038/s41419-024-07249-6)

Unedited blot and gel images

Figure S2B

Aβ (Soluble)

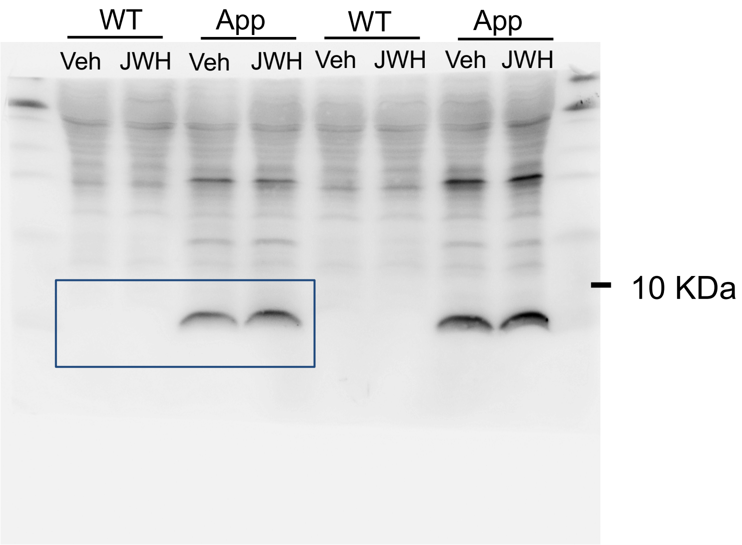

β-actin

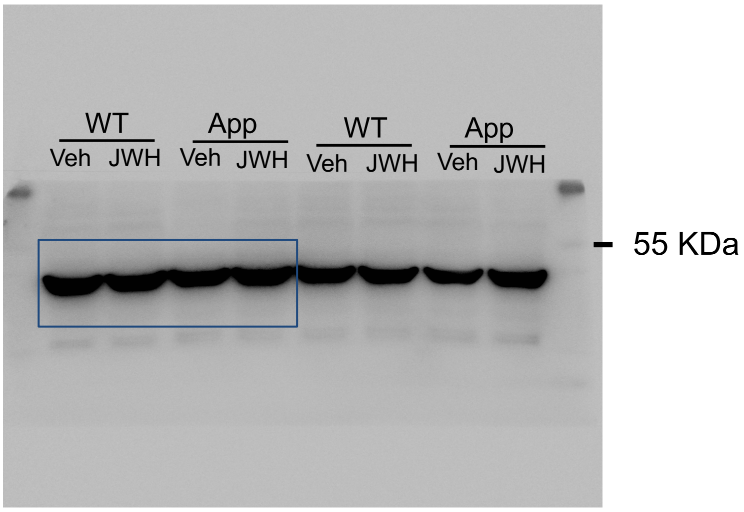

Figure S2D

Aβ (Insoluble)

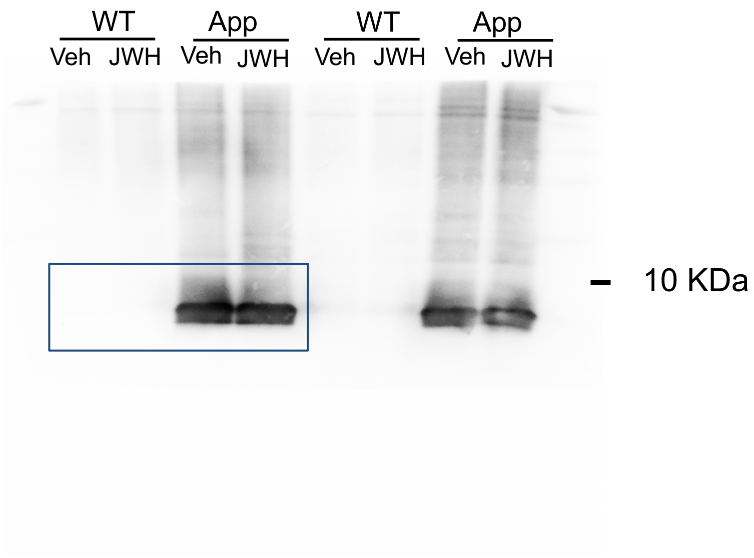

SYPRO Ruby staining

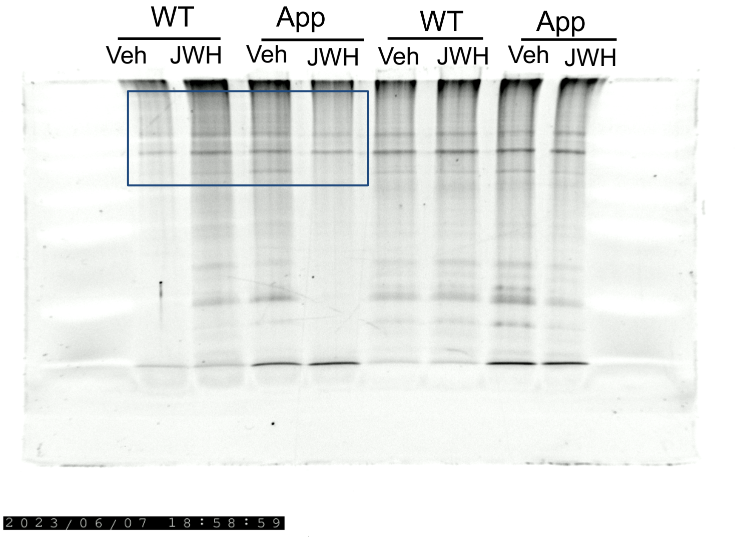

Supplement: Supplementary file 4 — Unedited blot and gel images [file 41419_2024_7249_MOESM4_ESM.pdf]
